# Cancer Incidence Trends in Successive Social Generations in the US

**DOI:** 10.1001/jamanetworkopen.2024.15731

**Published:** 2024-06-10

**Authors:** Philip S. Rosenberg, Adalberto Miranda-Filho

**Affiliations:** 1 Division of Cancer Epidemiology and Genetics, Biostatistics Branch, National Cancer Institute, Rockville, Maryland

## Abstract

**Importance:**

The incidence of some cancers in the US is increasing in younger age groups, but underlying trends in cancer patterns by birth year remain unclear.

**Objective:**

To estimate cancer incidence trends in successive social generations.

**Design, Setting, and Participants:**

In this cohort study, incident invasive cancers were ascertained from the Surveillance, Epidemiology, and End Results (SEER) program’s 13-registry database (November 2020 submission, accessed August 14, 2023). Invasive cancers diagnosed at ages 35 to 84 years during 1992 to 2018 within 152 strata were defined by cancer site, sex, and race and ethnicity.

**Exposure:**

Invasive cancer.

**Main Outcome and Measures:**

Stratum-specific semiparametric age-period-cohort (SAGE) models were fitted and incidence per 100 000 person-years at the reference age of 60 years was calculated for single-year birth cohorts from 1908 through 1983 (fitted cohort patterns [FCPs]). The FCPs and FCP incidence rate ratios (IRRs) were compared by site for Generation X (born between 1965 and 1980) and Baby Boomers (born between 1946 and 1964).

**Results:**

A total of 3.8 million individuals with invasive cancer (51.0% male; 8.6% Asian or Pacific Islander, 9.5% Hispanic, 10.4% non-Hispanic Black, and 71.5% non-Hispanic White) were included in the analysis. In Generation X vs Baby Boomers, FCP IRRs among women increased significantly for thyroid (2.76; 95% CI, 2.41-3.15), kidney (1.99; 95% CI, 1.70-2.32), rectal (1.84; 95% CI, 1.52-2.22), corpus uterine (1.75; 95% CI, 1.40-2.18), colon (1.56; 95% CI, 1.27-1.92), and pancreatic (1.39; 95% CI, 1.07-1.80) cancers; non-Hodgkins lymphoma (1.40; 95% CI, 1.08-1.82); and leukemia (1.27; 95% CI, 1.03-1.58). Among men, IRRs increased for thyroid (2.16; 95% CI, 1.87-2.50), kidney (2.14; 95% CI, 1.86-2.46), rectal (1.80; 95% CI, 1.52-2.12), colon (1.60; 95% CI, 1.32-1.94), and prostate (1.25; 95% CI, 1.03-1.52) cancers and leukemia (1.34; 95% CI, 1.08-1.66). Lung (IRR, 0.60; 95% CI, 0.50-0.72) and cervical (IRR, 0.71; 95% CI, 0.57-0.89) cancer incidence decreased among women, and lung (IRR, 0.51; 95% CI, 0.43-0.60), liver (IRR, 0.76; 95% CI, 0.63-0.91), and gallbladder (IRR, 0.85; 95% CI, 0.72-1.00) cancer and non-Hodgkins lymphoma (IRR, 0.75; 95% CI, 0.61-0.93) incidence decreased among men. For all cancers combined, FCPs were higher in Generation X than for Baby Boomers because gaining cancers numerically overtook falling cancers in all groups except Asian or Pacific Islander men.

**Conclusions and Relevance:**

In this model-based cohort analysis of incident invasive cancer in the general population, decreases in lung and cervical cancers in Generation X may be offset by gains at other sites. Generation X may be experiencing larger per-capita increases in the incidence of leading cancers than any prior generation born in 1908 through 1964. On current trajectories, cancer incidence could remain high for decades.

## Key Points

**Question:**

Is cancer incidence in successive social generations in the US slowing or growing?

**Findings:**

In this cohort study of 3.8 million patients with cancer ascertained by the Surveillance, Epidemiology, and End Results Program, members of Generation X born between 1965 and 1980 have been experiencing larger per-capita increases in the incidence of leading cancers combined than any prior generation born between 1908 and 1964.

**Meaning:**

These findings suggest that based on current trajectories, cancer incidence in the US might remain high for decades.

## Teaser

This cohort study uses Surveillance, Epidemiology, and End Results program registry data to estimate cancer incidence trends in successive social generations in the US.

## Introduction

Is cancer incidence in the US—ie, the number of newly diagnosed cases per capita per year—slowing or growing? Notably, while the incidence of certain cancers is declining,^1^ others are increasing in younger age groups (aged <50 years).^2^ These temporal patterns encompass a diversity of neoplasms and vary over time by demographic factors, including age, sex, and race and ethnicity. A more fundamental question is: are we collectively experiencing lower cancer incidence as we age than the generation of our parents?

Analyses that focus on birth years and social generations can provide insights into cancer prevention and health care accessibility and highlight persistent demographic and socioeconomic inequalities that span generations. Nevertheless, tracking the history of cancer in the US is challenging because cancers evolve over the lifetimes of individuals,^3^ yet the observational period of contemporary cancer registries spans at most a few decades.^4^ Hence, we must reconstruct the longitudinal history of cancer in the population from a time-limited series of cross-sectional observations. To understand the overarching trends, it is imperative to assemble these snapshots into a cohesive longitudinal narrative for a variety of cancer types in diverse demographic groups.

With the use of new statistical methods,^5,6^ we can now obtain single-year reconstructions of cancer incidence by age, period, and birth cohort with unprecedented precision. Our goal is to model the shifting landscape of cancer from one birth year to the next for leading cancers in men and women by race and ethnicity. From this ensemble, we hope to glean insights into the overall trajectory of cancer in the US from the Greatest Generation (born from 1908 through 1927) through Generation X (born from 1965 through 1980).

## Methods

### Cancer Incidence

Our cohort study was based on publicly available, deidentified data and, therefore, the National Cancer Institute institutional review board determined it exempt from review and informed consent. This study follows the Strengthening the Reporting of Observational Studies in Epidemiology (STROBE) reporting guideline.

We collected case and person-years at risk data from the Surveillance, Epidemiology, and End Results (SEER) program’s 13-registery database using SEER*Stat, version 8.4.0. Data ascertained for the November 2020 submission were retrieved on August 14, 2023 (eTable 1 in Supplement 1). Using these data, we assembled 152 rate matrix Lexis diagrams^7^ covering 50 single years of age (35-84 years) and 27 calendar years (1992-2018), spanning 76 single-year birth cohorts (1908-1983) for 21 leading invasive cancers in women and men within 4 racial and ethnic categories: Asian or Pacific Islander, Hispanic, non-Hispanic Black, and non-Hispanic White. The definitions of race and ethnicity follow the criteria established by the SEER program.^8^ In brief, the SEER program uses an algorithm to recode detailed race and origin variables in the SEER incidence data. The 21 leading invasive cancers were esophageal, stomach, gallbladder, liver, pancreatic, colon, rectal, kidney, bladder, leukemia, non-Hodgkin lymphoma (NHL), myeloma, brain and central nervous system (hereafter, brain), thyroid, lung, melanoma of skin (hereafter, melanoma), breast, ovarian, corpus uterine (hereafter, corpus), cervix uterine (hereafter, cervical), and prostate (eTable 2 in Supplement 1).

### Statistical Analysis

We analyzed each Lexis diagram using semiparametric age-period-cohort (SAGE) analysis.^6^ The basic idea is to denoise the observed Lexis diagrams upfront by using a contemporary nonparametric procedure,^5^ then fit the new age-period-cohort (APC) model to the smoothed Lexis diagrams.^9^ The new APC model contains parameters that describe how expected rates vary as a function of birth cohort, accounting for age and calendar period effects. We used the model parameters to estimate the fitted cohort pattern (FCP). The FCP estimates the absolute incidence of a cancer at an arbitrary reference age (here, 60 years) for each birth year in the Lexis diagram.^9^ Additional details are provided in the eMethods in Supplement 1.

To further characterize FCP features, we fitted Joinpoint models^10^ to the FCPs, allowing up to 5 segments, each with 10 or more birth years. We also computed mean FCPs and corresponding variances for the following social generations: Greatest Generation (1908-1927), Silent Generation (1928-1945), Baby Boomers (1946-1964), and Generation X (1965-1980). We had too few data points to produce estimates for Millennials (1981-1996).

To compare and contrast mean FCP values across generations, we fitted log-linear models to the generational differences in site-specific FCPs among men and women separately for the Silent vs Greatest Generations, Baby Boomers vs the Silent Generation, and Generation X vs Baby Boomers. We adjusted for race and ethnicity with non-Hispanic White as the reference group and weighted the data inversely with the estimated variance of each difference.

The SEER registries do not record the parents’ birth year for each cancer case. However, on average, the parents of Generation X are Baby Boomers and members of the Silent Generation, and the parents of Baby Boomers are members of the Silent and Greatest Generations. To see this assumption, subtract 20 through 29 years (mean maternal age at birth during our study period^11^) from the end points of each generation, yielding 1917-1944 for Baby Boomers, which straddles the Greatest and Silent Generations (eg, 1917-1927 and 1928-1944, respectively), and 1936-1960 for Generation X, which straddles the Silent Generation and Baby Boomers (eg, 1936-1945 and 1946-1960, respectively). Similarly, the proxy parents of the Millennial generation (1981-1996) straddle the Baby Boomers and Generation X (eg, 1952-1964 and 1965-1976, respectively). In our study, we make no adjustments for multiple testing.

## Results

In total, we analyzed 3.8 million cases of incident cancer occurring over 521 million person-years (eTable 2 in Supplement 1). Overall, 51.0% of individuals were male (compared with 49.0% female) and 71.5% were non-Hispanic White (compared with 8.6% Asian or Pacific Islander, 9.5% Hispanic, and 10.4% non-Hispanic Black).

### SAGE Analysis

The SAGE analysis produced optimally smoothed single-year estimates of incidence for 80 female strata (observed Lexis diagrams, eFigure 1 in Supplement 1; smoothed APC fitted rates, eFigure 2 in Supplement 1) and 72 male strata (observed, eFigure 3 in Supplement 1; smoothed, eFigure 4 in Supplement 1). For the majority, lack of fit (LOF) (females, eFigure 5 in Supplement 1; males, eFigure 6 in Supplement 1) was negligible or small relative to the corresponding annual fluctuations (females, eFigure 7 in Supplement 1; males, eFigure 8 in Supplement 1). The most prominent LOF was observed for liver, rectal, and brain cancers and melanoma in females and liver, brain, thyroid, and prostate cancers in males. Time trends by age (local drifts) were significant in all strata (females, eFigure 9 in Supplement 1; males, eFigure 10 in Supplement 1). As shown previously,^6^ LOF had little effect on the local drifts for the Lexis diagrams included in our study. Hence, the model fits appear adequate.^6^

### FCPs

**Figure 1.**
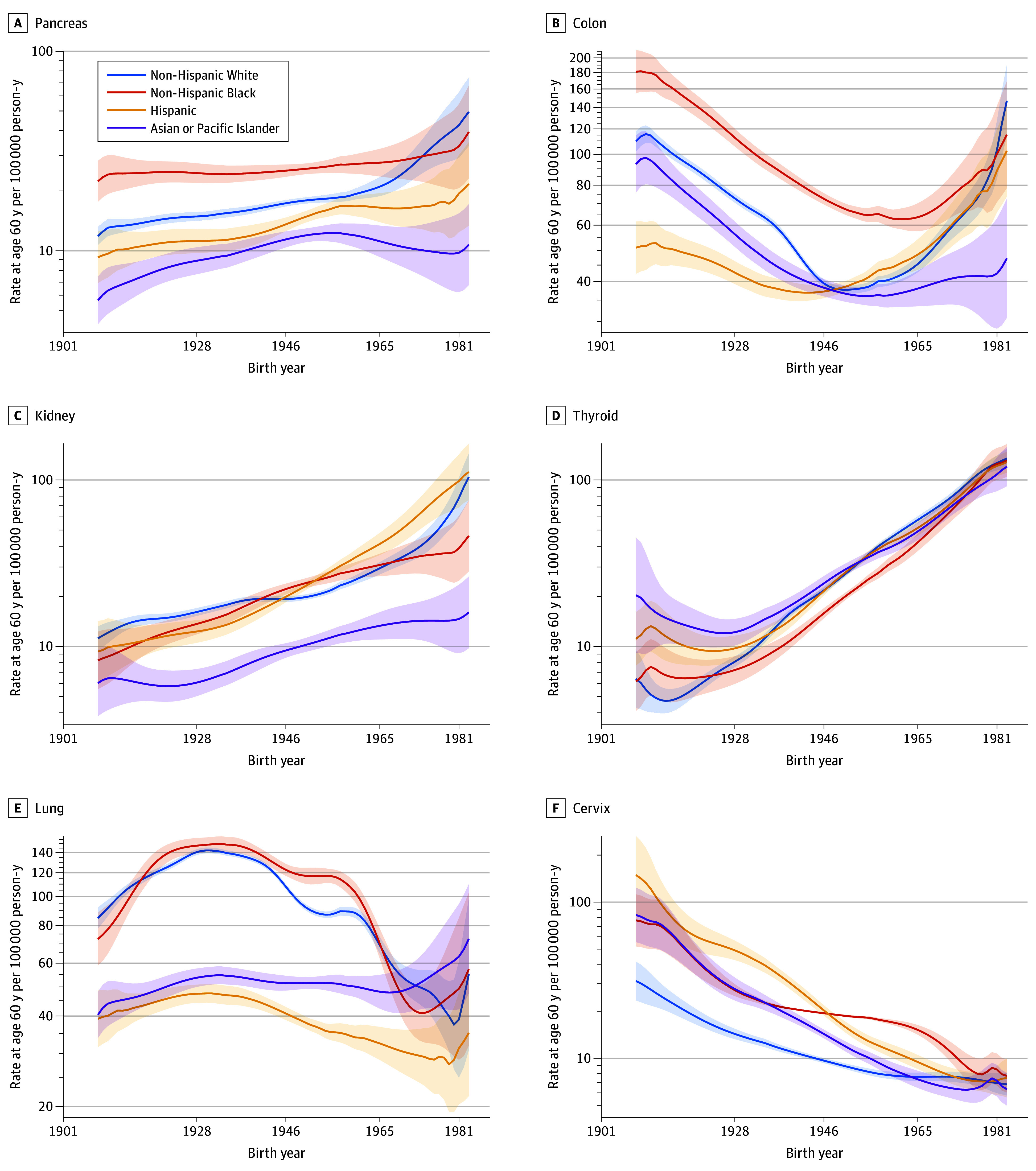
Fitted Cohort Patterns by Cancer Site and Race and Ethnicity: Females Estimated incidence per 100 000 person-years at age 60 years is shown by birth year (1908-1983). All curves are on the log_10_ scale. These and additional curves are shown in eFigure 11 in Supplement 1. Tick marks on the x-axis indicate start years for consecutive social generations: 1928-1945, Silent Generation; 1946-1964, Baby Boomers; 1965-1980, Generation X. Shaded areas indicate 95% CIs.

eFigure 11 in Supplement 1 presents FCPs by cancer site for women by race and ethnicity. Six of these FCPs, including pancreatic, colon, kidney, thyroid, lung, and cervical, are also shown in Figure 1A through F, respectively. The statistical efficiency of the SAGE analysis yielded partially or fully nonoverlapping curves within most FCPs; the conclusions below were obtain by visual inspection.

Differences by race and ethnicity strongly depended on birth cohort. There were marked declines in cervical FCPs across all racial and ethnic groups. Incidence rates were highest among the Greatest Generation in non-Hispanic Black women for esophageal, pancreatic, and colon cancers and myeloma; in Hispanic women for gallbladder cancer; in Asian or Pacific Islander women for stomach and liver cancers; and in non-Hispanic White women for bladder, breast, ovarian, and corpus cancers and leukemia, NHL, and melanoma. Patterns by race and ethnicity were similar for the Silent Generation. Patterns changed with the Baby Boomers. Notable changes included convergence of esophageal cancers in non-Hispanic Black and non-Hispanic White women; steep declines for stomach cancer in Asian or Pacific Islander women; and steep increases in corpus cancer for Asian or Pacific Islander, Hispanic, and non-Hispanic Black women. In Generation X women, the FCPs consistently increased for liver, colon, rectal, kidney, thyroid, and corpus cancers (Figure 1; eFigure 11 in Supplement 1). Estimates of the corresponding estimated annual percentage changes (EAPCs) of the FCPs obtained by Joinpoint analysis are shown in eFigure 12 in Supplement 1.

**Figure 2.**
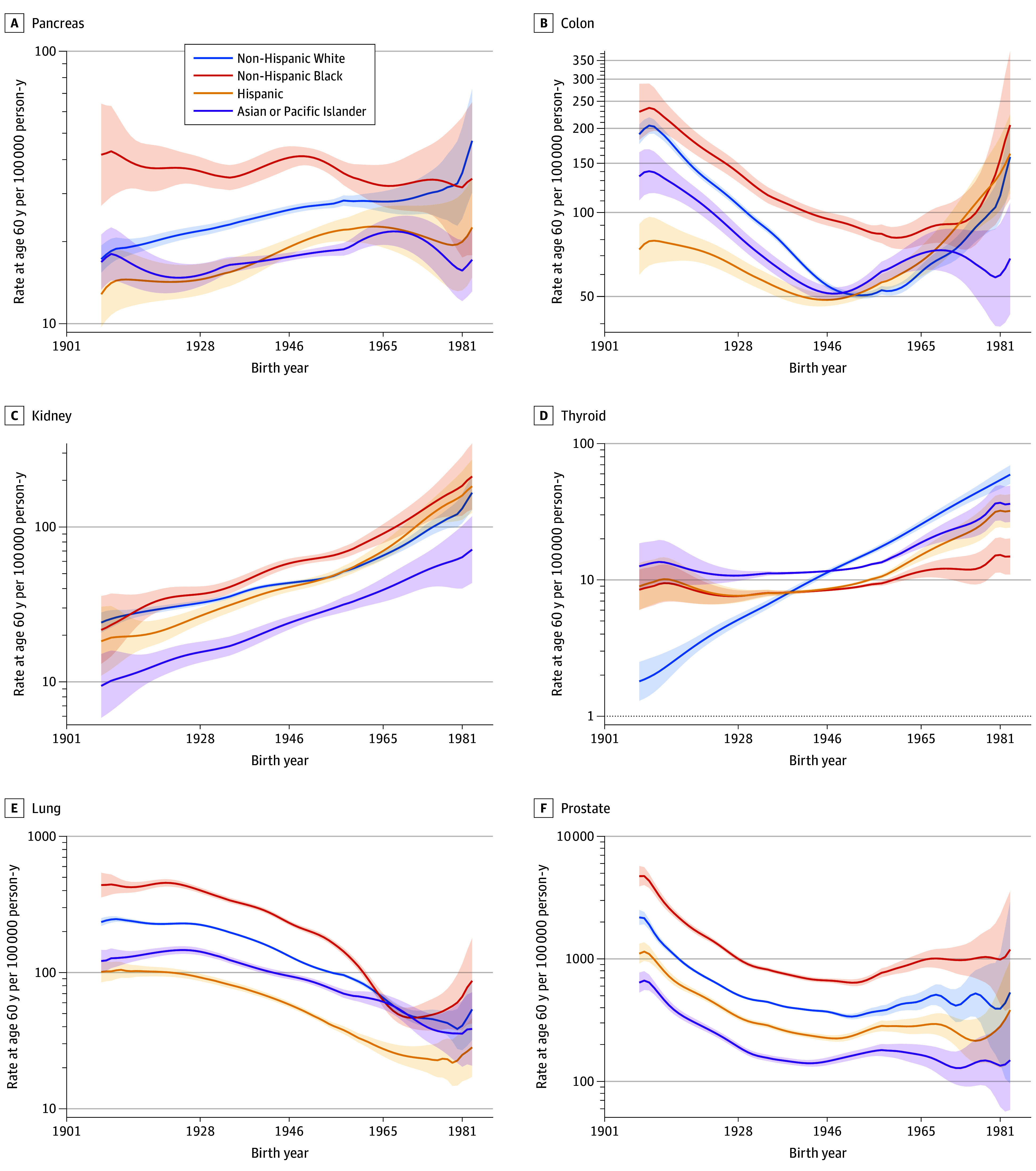
Fitted Cohort Patterns by Cancer Site and Race and Ethnicity: Males Estimated incidence per 100 000 person-years at age 60 years is shown by birth year (1908-1983). All curves are on the log_10_ scale. These and additional curves are shown in eFigure 13 in Supplement 1. Tick marks on the x-axis indicate start years for consecutive social generations: 1928-1945, Silent Generation; 1946-1964, Baby Boomers; 1965-1980, Generation X. Shaded areas indicate 95% CIs.

eFigure 13 in Supplement 1 presents FCPs by cancer site for men. Six of these FCPs, including pancreatic, colon, kidney, thyroid, lung, and prostate, are also shown in Figure 2A through F, respectively. For thyroid cancer, increases by cohort were slower in Asian or Pacific Islander, Hispanic, and non-Hispanic Black men compared with women, and peak incidence for lung cancer occurred earlier in men compared with women. The FCPs for prostate cancer were parallel across racial and ethnic groups, being highest in non-Hispanic Black and lowest in Asian or Pacific Islander men. In Generation X men, the FCPs were consistently increasing for colon, rectal, kidney, and thyroid cancers (Figure 2; eFigure 13 in Supplement 1). eFigure 14 in Supplement 1 provides the corresponding estimated EAPCs.

**Figure 3.**
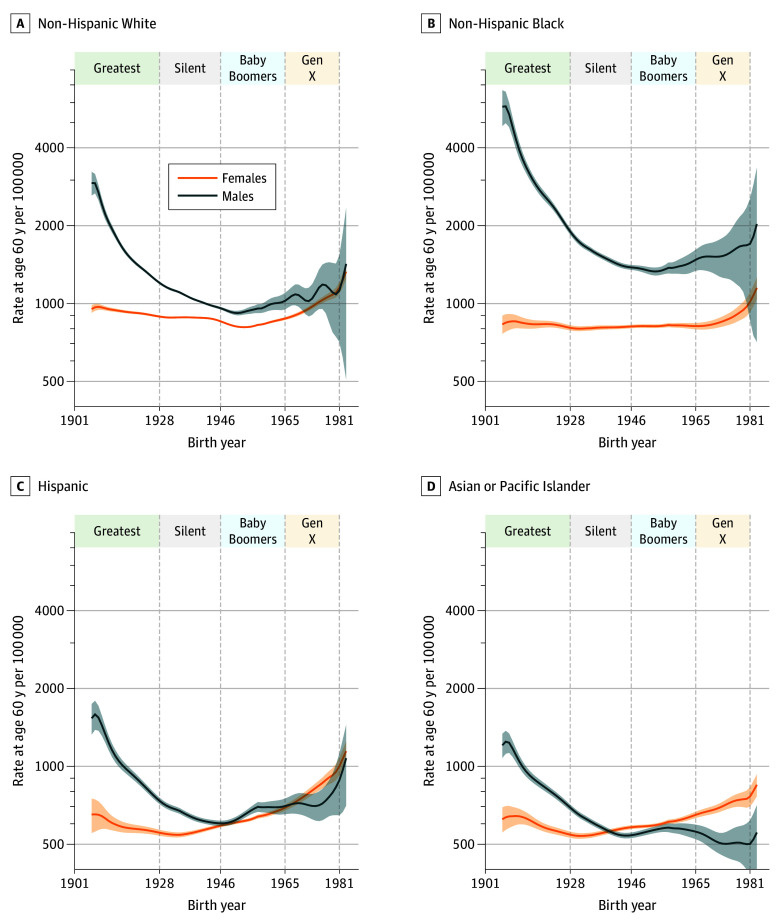
Fitted Cohort Patterns for Leading Cancers Combined Gen X indicates Generation X. Shaded areas indicate 95% CIs.

Figure 3 presents the sum of the cancer site–specific FCPs (20 sites in women and 18 in men) by sex and race and ethnicity. Among men, incidence at age 60 years declined through the Greatest and Silent Generations in all 4 racial and ethnic groups. Subsequently, incidence increased for the Baby Boomers. Except for Asian or Pacific Islander men, incidence continued to increase in Generation X.

In contrast, among women, incidence was comparatively stable among members of the Greatest and Silent Generations and then increased beginning with the Baby Boomers for non-Hispanic White individuals (Figure 3A), then the Silent Generation for Hispanic and Asian or Pacific Islander (Figure 3C and D) and Generation X for non-Hispanic Black individuals (Figure 3B). Within each racial and ethnic group, the large male excess in the Greatest Generation diminished in subsequent generations. By Generation X, Hispanic and non-Hispanic White women had a similar incidence to their male counterparts (Figure 3A and C), and Asian or Pacific Islander women had a higher incidence compared with their male counterparts (Figure 3D). In the non-Hispanic Black group, the male excess persisted.

### Successive Social Generations: Contrasts

eFigure 15 in Supplement 1 presents arrow plots of mean FCP values by cancer site for Generation X vs Baby Boomers stratified by sex and race and ethnicity. Within each stratum, the FCP for Generation X was higher than the corresponding FCP for Baby Boomers at some sites and lower at others. Similar heterogeneity was observed in arrow plots of Baby Boomers vs the Silent Generation (eFigure 16 in Supplement 1) and the Silent vs Greatest Generations (eFigure 17 in Supplement 1).

To synthesize these data, we estimated FCP incidence rate ratios (IRRs) by fitting log-linear models to the estimated FCPs in eFigures 15 to 17 in Supplement 1. For example, for Generation X vs Baby Boomer women, we synthesized the 80 arrows shown in panels eFigure 15A to D in Supplement 1. Each arrow, which is indexed by site and race and ethnicity, contributed 1 difference or IRR to the modeled data. The estimated variances of the differences were used as weights.

**Figure 4.**
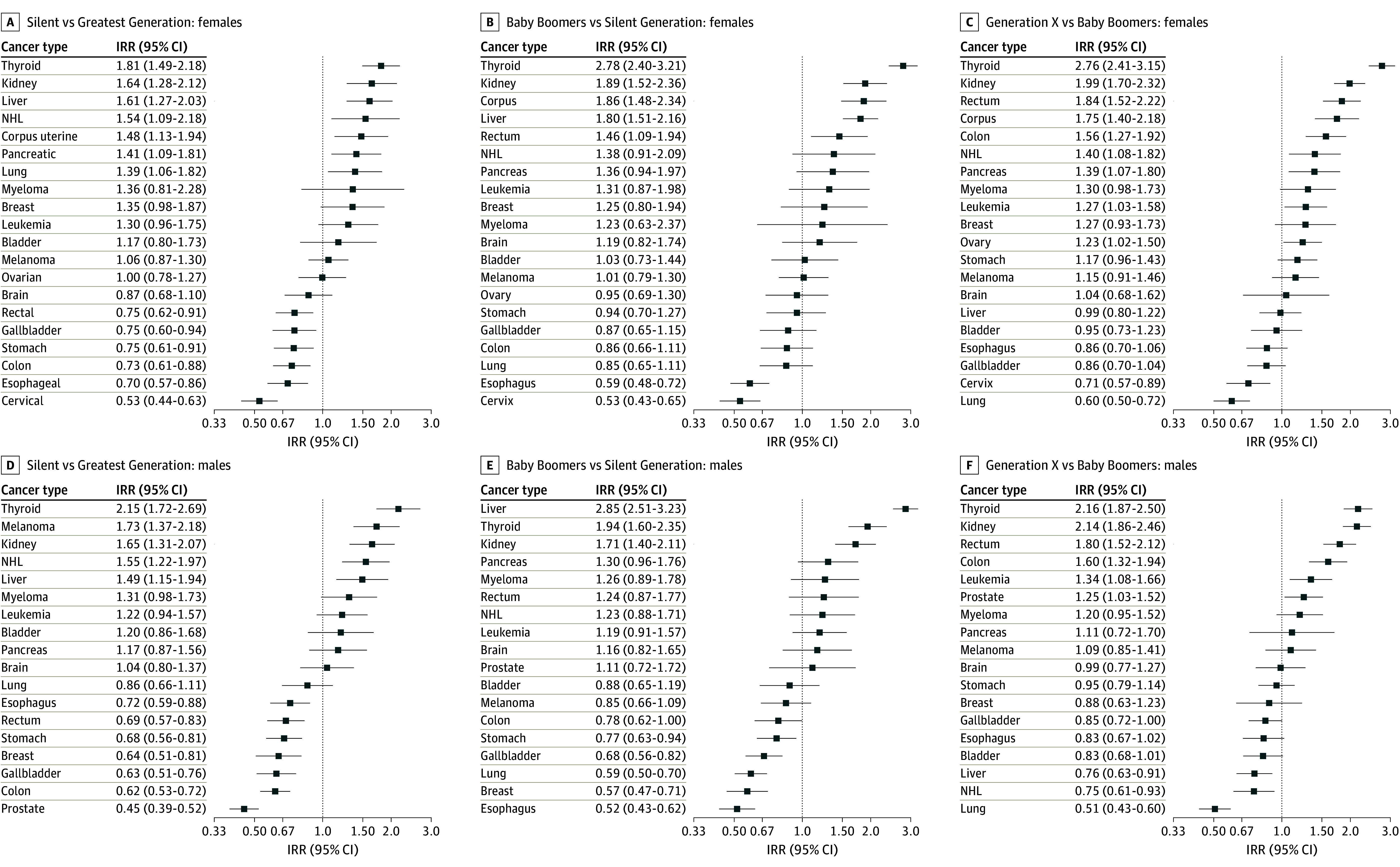
Incidence by Cancer Site in Successive Generations Fitted cohort pattern curves by cancer site are adjusted for race and ethnicity as described in the Methods, plotted on a natural log scale, and sorted from largest (top) to smallest (bottom). IRR indicates incidence rate ratio; NHL, non-Hodgkins lymphoma.

Figure 4 presents forest plots of the site-specific IRRs between successive social generations adjusted for race and ethnicity in women and men. Point estimates are shown for non-Hispanic White individuals. Estimates for other racial and ethnic groups are similar (eFigure 18 in Supplement 1). Site-specific IRRs varied markedly by generation except for the brain.

The incidence of many cancers increased significantly in members of Generation X vs Baby Boomers (Figure 4). Among women, increases were observed for the following cancers: thyroid (IRR, 2.76, 95% CI, 2.41-3.15), kidney (IRR, 1.99; 95% CI, 1.70-2.32), rectal (IRR, 1.84; 95% CI, 1.52-2.22), corpus (IRR, 1.75; 95% CI, 1.40-2.18), colon (IRR, 1.56; 95% CI, 1.27-1.92), NHL (IRR, 1.40, 95% CI 1.08-1.82), pancreatic (IRR, 1.39; 95% CI, 1.07-1.80), and leukemia (IRR, 1.27; 95% CI, 1.03-1.58). Among men, increases were observed for the following cancers: thyroid (IRR, 2.16; 95% CI, 1.87-2.50), kidney (IRR, 2.14; 95% CI, 1.86-2.46), rectal (IRR, 1.80; 95% CI, 1.52-2.12), colon (IRR, 1.60; 95% CI, 1.32-1.94), leukemia (IRR, 1.34; 95% CI, 1.08-1.66), and prostate (IRR, 1.25; 95% CI, 1.03-1.52). Other cancers decreased, including lung (IRR, 0.60; 95% CI, 0.50-0.72) and cervical (IRR, 0.71; 95% CI, 0.57-0.89) among women and lung (IRR, 0.51; 95% CI, 0.43-0.60), NHL (IRR, 0.75; 95% CI, 0.61-0.93), liver (IRR, 0.76; 95% CI, 0.63-0.91), and gallbladder (IRR, 0.85; 95% CI, 0.72-1.00) among men.

**Figure 5.**
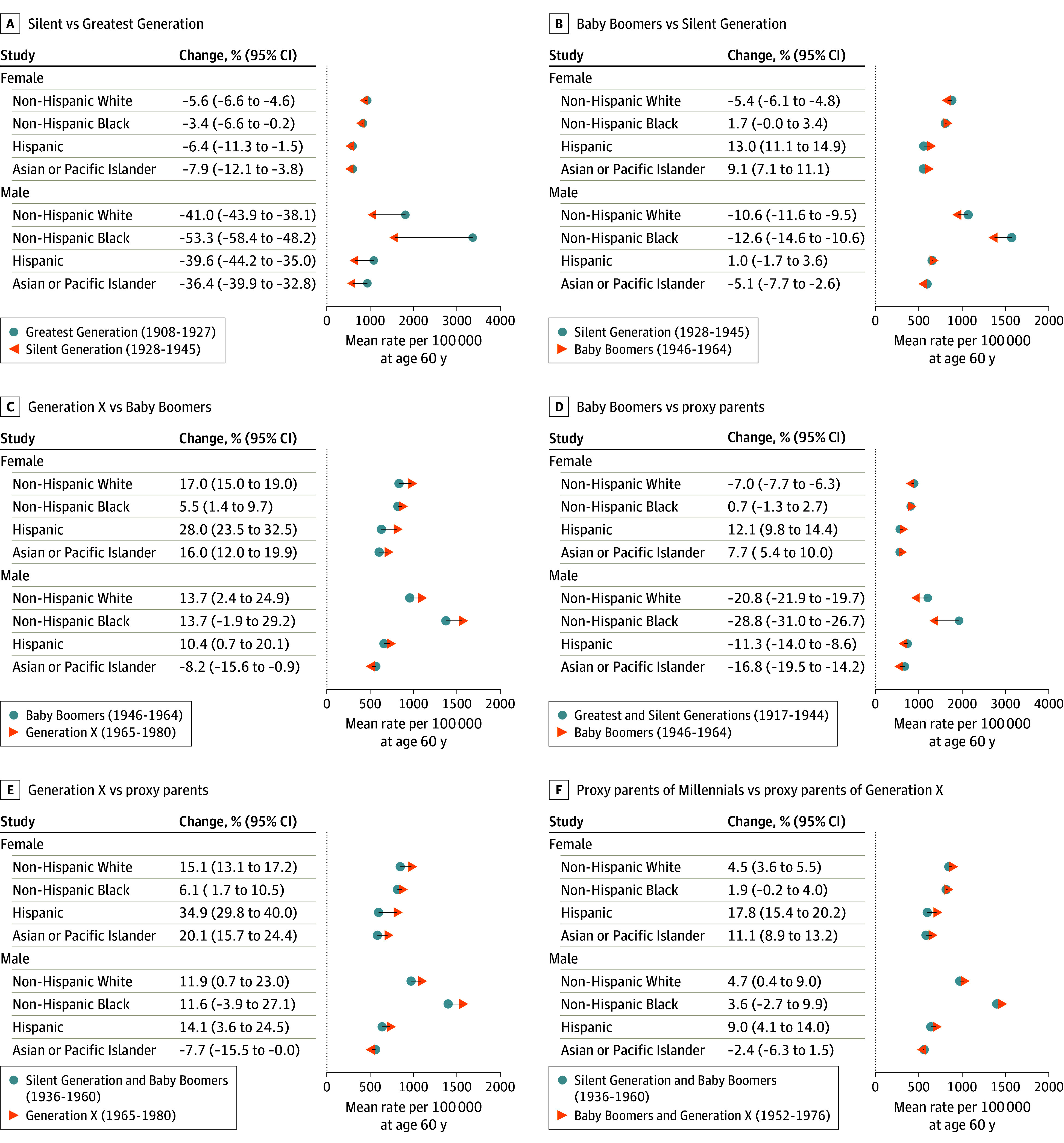
Changes in Leading Cancer Incidence at Age 60 Years per 100 000 Person-Years in Successive Generations and Proxy Parents by Sex and Race and Ethnicity Arrow plots indicate magnitude and direction of change from older to younger generations. Corresponding percentage changes and 95% CIs are also graphed in eFigure 19 in Supplement 1.

Figure 5 summarizes incidence at age 60 years for leading cancers combined, averaged over birth years within successive generations. eFigure 19 in Supplement 1 plots the corresponding percentage changes. Comparing the Silent vs Greatest Generations (Figure 5A), combined incidence decreased significantly in both sexes and all 4 racial and ethnic groups. The decreases were greater for men compared with women. For example, incidence decreased by 41.0% (95% CI, −43.9% to −38.1%) in non-Hispanic White men vs 5.6% (95% CI, −6.6% to −4.6%) in non-Hispanic White women. In contrast, comparing Baby Boomers with the Silent Generation (Figure 5B), combined incidence had a mixed pattern, decreasing in some groups, eg, by 5.4% (95% CI, −6.1% to −4.8%) and 10.6% (95% CI, −11.6% to −9.5%), respectively, in non-Hispanic White women and men, but increasing in others, eg, by 13.0% (95% CI, 11.1%-14.9%) among Hispanic women.

Comparing Generation X with Baby Boomers (Figure 5C), combined incidence increased in all groups except for Asian or Pacific Islander men. The increases ranged from 5.5% (95% CI, 1.4%-9.7%) in non-Hispanic Black women to 28.0% (95% CI, 23.5%-32.5%) in Hispanic women. The increases ranged from 10.4% (95% CI, 0.7%-20.1%) in Hispanic men to 13.7% (95% CI, 2.4%-24.9%) in non-Hispanic White men. The increase among non-Hispanic Black men was 13.7% (95% CI, −1.9% to 29.2%). Incidence decreased by 8.2% (95% CI, −15.6% to −0.9%) among Asian or Pacific Islander men. Except for Asian or Pacific Islander men, these increases were larger than any observed in prior social generations (eg, point estimates in Figure 5C are larger than corresponding point estimates in Figure 5A and B). In absolute terms, non-Hispanic Black men in Generation X had the highest combined incidence at 1561 cases per 100 000 person-years (95% CI, 1493-1629 cases per 100 000 person-years), while Asian or Pacific Islander men had the lowest at 519 cases per 100 000 person-years (95% CI, 474-563 cases per 100 000 person-years).

### Baby Boomers, Generation X, and Proxy Parents

Male Baby Boomers had a lower combined incidence than their proxy parents (Greatest and Silent Generation birth cohorts from 1917 to 1944) (Figure 5D), with the greatest reduction of 28.8% (95% CI, −31.0 to −26.7%) in non-Hispanic Black men. The decreases among men were statistically significant in the other 3 racial and ethnic groups (Asian or Pacific Islander, −16.8% [95% CI, −19.5% to −14.2%]; Hispanic, −11.3% [95% CI, −14.0% to −8.6%]; non-Hispanic White, −20.8% [95% CI, −21.9% to −19.7%]). Female Baby Boomers had a mixed pattern: non-Hispanic White women, 7.0% lower (95% CI, −7.7% to −6.3%); essentially unchanged in non-Hispanic Black women, 0.7% higher (95% CI, −1.3% to 2.7%); Hispanic women, 12.1% higher (95% CI, 9.8%-14.4%); Asian or Pacific Islander women, 7.7% higher (95% CI, 5.4%-10.0%).

In contrast, members of Generation X had a higher combined incidence than their proxy parents (Silent Generation and Baby Boomers birth cohorts from 1936 to 1960) (Figure 5E) in all demographic groups except for Asian or Pacific Islander men. These increases were statistically significant in all 4 racial and ethnic groups among women (Asian or Pacific Islander, 20.1% [95% CI, 15.7%-24.4%]; Hispanic, 34.9% [95% CI, 29.8%-40.0%]; non-Hispanic Black, 6.1% [95% CI, 1.7%-10.5%]; non-Hispanic White, 15.1% [95% CI, 13.1%-17.2%]) and in Hispanic (14.1%; 95% CI, 3.6%-24.5%) and non-Hispanic White (11.9%; 95% CI, 0.7%-23.0%) men. Similarly, the proxy parents of the Millennials (Baby Boomer and Generation X birth cohorts from 1952 to 1976) also had higher cancer incidence than the proxy parents of Generation X in all demographic groups except Asian or Pacific Islander males (Figure 5F). The increases were statistically significant in Asian or Pacific Islander (11.1%; 95% CI, 8.9%-13.2%), Hispanic (17.8%; 95% CI, 15.4%-20.2%), and non-Hispanic White (4.5%; 95% CI, 3.6%-5.5%) women and in Hispanic (9.0%; 95% CI, 4.1%-14.0%) and non-Hispanic White (4.7%; 95% CI, 0.4%-9.0%) men.

## Discussion

In this cohort study, we asked whether we, collectively, are experiencing lower cancer incidence as we age than our parents. Using SAGE analysis, we were able to reconstruct the cancer experience of social generations from the Greatest Generation through Generation X and use selected birth years from the 2 preceding social generations as a proxy for the corresponding parental generation. To arrive at an overall conclusion, we used a simple summary measure: the incidence of leading cancers combined (20 sites in women and 18 sites in men).

For Baby Boomers (1946-1964) vs their proxy parents (1917-1944), the answer was yes for men (ie, progress) and mixed for women (Figure 5D). For Generation X (1965-1980) vs their proxy parents (1936-1960), the answer was no in all groups except for Asian or Pacific Islander males (Figure 5E).

The increases in cancer incidence in members of Generation X vs their proxy parents were substantial, especially among Hispanic women (a 34.9% increase) and men (a 14.1% increase). In contrast, the corresponding increases among non-Hispanic White women and men were 15.1% and 11.9%, respectively. We obtained similar results when we compared the Generation X to Baby Boomer social generations (Figure 5C). Furthermore, between the Greatest Generation and Generation X, the historical male cancer excess narrowed (non-Hispanic Black and non-Hispanic White men), declined to parity (Hispanic men), or reversed (Asian or Pacific Islander men) (Figure 3).

Our conclusions are more concerning than previously reported increases in cancer incidence in younger age groups.^2,12^ Those results, based on local drifts, describe the slope of the FCP curve in consecutive blocks of P birth years, where P is the total number of calendar years in the analysis (eg, *P* = 19 in Sung et al^2^; *P* = 27 in our analyses).^9^ For this reason, local drifts provide lagging and conservative indicators of changes from one birth year to the next. Furthermore, using SAGE analysis, we were able to estimate absolute FCP values for single-year birth cohorts and consecutive changes in incidence per birth year vs the 10-year overlapping cohort relative risks reported by Sung et al.^2^

The substantial increases we identified in Generation X vs both the Baby Boomers and their proxy parents surprised us. Numerous preventable causes of cancer have been identified.^13^ Cancer control initiatives have led to substantial declines in tobacco consumption.^14^ Screening is well accepted for precancerous lesions of the colon, rectum, cervix, uterus, and breast.^15^ However, other suspected carcinogenic exposures are increasing.^16,17,18^

Unfortunately, as shown in our detailed comparative analysis of Generation X vs Baby Boomers, gaining cancers have numerically overtaken falling cancers. Among Generation X women, statistically significant declines in lung and cervical cancers have been overtaken by significant increases in thyroid, kidney, rectal, corpus, colon, pancreatic, and ovarian cancer and NHL and leukemia (Figure 4C). Among Generation X men, declines in lung, liver, and gallbladder cancers and NHL have been overtaken by gains in thyroid, kidney, rectal, colon, and prostate cancers and leukemia (Figure 4F).

Some portion of these increases can be attributed to rising obesity rates^19^ and increasingly sedentary lifestyles.^20,21,22,23^ Another portion might be explained by changes in cancer registry policies and *International Statistical Classification of Diseases and Related Health Problems, 10th Revision* (World Health Organization) classifications,^24^ leading to inclusion of relatively indolent lesions in more recent periods that might not have been diagnosed as cancer in earlier periods. Furthermore, radiologic diagnoses have become more common following widespread deployment of sophisticated medical imaging technologies,^25^ especially for thyroid^26,27^ and kidney^28,29^ cancers. We chose not to exclude any leading cancer site from our summaries because our granular estimates are freely available (eFigures 15-17 in Supplement 1).

Our results beg the question of what the cancer experience may be like among the 72 million Millennials (1981-1996) when they enter their 40s, 50s, and 60s. On one hand, our analysis shows that the proxy parents of the Millennials are experiencing as much or more cancer than the proxy parents of Generation X (Figure 5F). This increase is concerning because of shared cancer-predisposing lifestyle factors and exposures. On the other hand, thanks to the global investment in cancer research, there are tremendous opportunities to prospectively reduce the Millennials’ future cancer burden.

The American Cancer Society,^30^ Centers for Disease Control and Prevention,^31^ and World Health Organization^13^ advocate a series of preventive actions to diminish cancer risks. These include reducing tobacco and alcohol use, increasing physical activity, improving dietary habits, and promoting breastfeeding. These recommendations can also reduce heart disease^32^ and cognitive decline.^33^

Unfortunately, universal implementation of these recommendations in the US is a work in progress. The Black-to-White cancer mortality gap narrowed following passage of the Patient Protection and Affordable Care Act.^34,35^ However, income inequality,^36^ underinsurance,^37,38,39^ food swamps and deserts,^40,41^ deficits in the built environment,^42^ and other factors make it difficult for everyone to eat healthy and stay active.^30^ Taken together, these findings indicate that for many people in the US, a healthy lifestyle remains, to various degrees, an unattainable privilege rather than a fundamental right. The extent to which lifestyle disparities explain rising generational cancer rates in our data and falling life expectancies in other studies^43^ is unclear and, in our view, merits further study.

### Limitations

Our study has 2 major limitations. First, the numbers of less common cancers in the SEER 13-registry database among Asian or Pacific Islander, Hispanic, and non-Hispanic Black men and women are limited, especially for esophageal and gallbladder cancers and melanoma. Second, our conclusions derive from modeling. Even so, we believe that our detailed analysis of 3.8 million individuals with invasive cancers in 152 distinct strata mitigated many potential biases. Furthermore, at most cancer sites, the birth cohort effects were substantial, and the LOF was relatively small. For this reason, we believe that it is appropriate to draw conclusions from our FCP estimates. However, it is important to appreciate that the FCP incorporates backward projection for older cohorts and forward projection for younger cohorts; in other words, it is very much a model-based quantity.

## Conclusions

The models in this cohort study suggest that Generation X is experiencing larger per-capita increases in the incidence of leading cancers combined than any prior generation born from 1908 through 1964. In addition, the rate of leading cancers appears to be as high or higher in the proxy parents of the Millennials than the proxy parents of Generation X. Therefore, if the Millennials’ cancer experience follows the estimated trajectory of their proxy parents, cancer incidence in the US could remain unacceptably high for decades to come.

## Notes

Supplement 1. **eMethods.** Statistical Methods **eTable 1.** Organ-Specific Site Code Used in the Classification of Cancer Sites Using SEER*Stat 8.4.0 **eTable 2.** Age-Standardized Incidence Rates and Cancer Cases by Sex, Race and Ethnicity, United States, 35-84 Years Old, 1992-2018 **eFigure 1.** Observed Rates in Females **eFigure 2.** APC Fitted Values in Females **eFigure 3.** Observed Rates in Males **eFigure 4.** APC Fitted Values in Males **eFigure 5.** Lack of Fit (LOF) in Females **eFigure 6.** Lack of Fit (LOF) in Males **eFigure 7.** Higher-Order Deviations vs Lack of Fit (LOF) in Females **eFigure 8.** Higher-Order Deviations vs Lack of Fit (LOF) in Males **eFigure 9.** Local Drifts in Females **eFigure 10.** Local Drifts in Males **eFigure 11.** Fitted Cohort Patterns (FCPs) by Cancer Site, Race, and Ethnicity: Females **eFigure 12.** Estimated Annual Percentage Change (EAPC) of the Fitted Cohort Pattern (FCP): Females **eFigure 13.** Fitted Cohort Patterns (FCPs) by Cancer Site, Race, and Ethnicity: Males **eFigure 14.** Estimated Annual Percentage Change (EAPC) of the Fitted Cohort Pattern (FCP): Males **eFigure 15.** Average Incidence at Age 60: Generation X vs Baby Boomers **eFigure 16.** Average Incidence at Age 60: Baby Boomers vs the Silent Generation **eFigure 17.** Average Incidence at Age 60: Silent vs Greatest Generations **eFigure 18.** Site-Adjusted Cancer Incidence Rate Ratios (IRRs) for Non-Hispanic Black, Hispanic, and Asian or Pacific Islander vs Non-Hispanic White by Sex and Social Generation **eFigure 19.** Percent Changes in Incidence of Leading Cancers at Age 60 Years per 100 000 Person-Years in Successive Generations

Supplement 2.
Data Sharing Statement
