# Supplementary material for: Cancer Incidence Trends in Successive Social Generations in the US
Source: JAMA Netw Open. 2024 Jun 10;7(6):e2415731. doi: 10.1001/jamanetworkopen.2024.15731 (PMC11165384; doi:10.1001/jamanetworkopen.2024.15731)
Supplement: Supplement 1. — eMethods. Statistical Methods eTable 1. Organ-Specific Site Code Used in the Classification of Cancer Sites Using SEER*Stat 8.4.0 eTable 2. Age-Standardized Incidence Rates and Cancer Cases by Sex, Race and Ethnicity, United States, 35-84 Years Old, 1992-2018 eFigure 1. Observed Rates in Females eFigure 2. APC Fitted Values in Females eFigure 3. Observed Rates in Males eFigure 4. APC Fitted Values in Males eFigure 5. Lack of Fit (LOF) in Females eFigure 6. Lack of Fit (LOF) in Males eFigure 7. Higher-Order Deviations vs Lack of Fit (LOF) in Females eFigure 8. Higher-Order Deviations vs Lack of Fit (LOF) in Males eFigure 9. Local Drifts in Females eFigure 10. Local Drifts in Males eFigure 11. Fitted Cohort Patterns (FCPs) by Cancer Site, Race, and Ethnicity: Females eFigure 12. Estimated Annual Percentage Change (EAPC) of the Fitted Cohort Pattern (FCP): Females eFigure 13. Fitted Cohort Patterns (FCPs) by Cancer Site, Race, and Ethnicity: Males eFigure 14. Estimated Annual Percentage Change (EAPC) of the Fitted Cohort Pattern (FCP): Males eFigure 15. Average Incidence at Age 60: Generation X vs Baby Boomers eFigure 16. Average Incidence at Age 60: Baby Boomers vs the Silent Generation eFigure 17. Average Incidence at Age 60: Silent vs Greatest Generations eFigure 18. Site-Adjusted Cancer Incidence Rate Ratios (IRRs) for Non-Hispanic Black, Hispanic, and Asian or Pacific Islander vs Non-Hispanic White by Sex and Social Generation eFigure 19. Percent Changes in Incidence of Leading Cancers at Age 60 Years per 100 000 Person-Years in Successive Generations [file jamanetwopen-e2415731-s001.pdf]

Supplemental Online Content

Rosenberg PS, Mirando-Filho A. Cancer incidence trends in successive social generations in the US. *JAMA Netw Open*. 2024;7(6):e2415731. doi:10.1001/jamanetworkopen.2024.15731

eMethods. Statistical Methods

eTable 1. Organ-Specific Site Code Used in the Classification of Cancer Sites Using SEER*Stat 8.4.0

eTable 2. Age-Standardized Incidence Rates and Cancer Cases by Sex, Race and Ethnicity, United States, 35-84 Years Old, 1992-2018

eFigure 1. Observed Rates in Females

eFigure 2. APC Fitted Values in Females

eFigure 3. Observed Rates in Males

eFigure 4. APC Fitted Values in Males

eFigure 5. Lack of Fit (LOF) in Females

eFigure 6. Lack of Fit (LOF) in Males

eFigure 7. Higher-Order Deviations vs Lack of Fit (LOF) in Females

eFigure 8. Higher-Order Deviations vs Lack of Fit (LOF) in Males

eFigure 9. Local Drifts in Females

eFigure 10. Local Drifts in Males

eFigure 11. Fitted Cohort Patterns (FCPs) by Cancer Site, Race, and Ethnicity: Females

eFigure 12. Estimated Annual Percentage Change (EAPC) of the Fitted Cohort Pattern (FCP): Females

eFigure 13. Fitted Cohort Patterns (FCPs) by Cancer Site, Race, and Ethnicity: Males

eFigure 14. Estimated Annual Percentage Change (EAPC) of the Fitted Cohort Pattern (FCP): Males

eFigure 15. Average Incidence at Age 60: Generation X vs Baby Boomers

eFigure 16. Average Incidence at Age 60: Baby Boomers vs the Silent Generation

eFigure 17. Average Incidence at Age 60: Silent vs Greatest Generations

eFigure 18. Site-Adjusted Cancer Incidence Rate Ratios (IRRs) for Non-Hispanic Black, Hispanic, and Asian or Pacific Islander vs Non-Hispanic White by Sex and Social Generation

eFigure 19. Percent Changes in Incidence of Leading Cancers at Age 60 Years per 100 000 Person-Years in Successive Generations

This supplemental material has been provided by the authors to give readers additional information about their work.

eMethods. Statistical Methods

Semi-Parametric Age-Period-Cohort (SAGE) Analysis

We analyzed each Lexis diagram using Semi-parametric age-period-cohort (SAGE) analysis. The basic idea is we denoise the observed Lexis diagrams up front using a contemporary nonparametric procedure, and then we fit the “new” age-period-cohort (APC) model to the smoothed Lexis diagrams. The New APC model contain parameters that describe how the expected rates vary as a function of age, period and birth cohort, assuming only that these effects are additive on the log scale. Informative combinations of these parameters, called “Estimable Functions” (EFs), are then calculated from the parameters of the New APC Model.

Because our contemporary nonparametric procedure provides a highly effective denoising tool, EFs obtained from the smoothed rates are more accurate (i.e., lower mean squared error). This allows us to obtain stable estimates from relatively sparse single-year Lexis diagrams, for example, Lexis diagrams for less common cancer types or numerically smaller race and ethnicity groups.

A schematic of the SAGE procedure is provided here. In brief, SAGE partitions the data into three pieces: APC Fitted Rates, Lack-of-Fit (LOF), and Pure Error.

APC Fitted Rates obtain from "Key Parameters" and "Higher-Order Terms". Together, these parameters represent the expected values of the rates as a function of age, times a function of period, times a function of cohort. The "Higher-Order Terms" describe year-over-year fluctuations in expected incidence over or above the underlying linear and quadratic trends by age, period, and birth cohort.

The model fit is adequate when the LOF is small relative to these Higher-Order Terms.

Fitted Cohort Pattern (FCP)

The Fitted Cohort Pattern (FCP) is a model-based estimate of the rate per 100,000 person-years at an arbitrary reference age (here, age 60 years) as a function of birth cohort, adjusted for period effects.

The FCP can be interpreted as a composite curve that includes backward projections for older cohorts and forward projections for younger cohorts.

For example, in the 29-year time frame of our study – we analyzed cases diagnosed between 1992 - 2018 – cohorts born 1932 – 1958 were directly observed at age 60 years; this window spans most of the Silent Generation and the first half of the Baby Boomers. However, cohorts born from 1908 – 1931 were older than age 60 years at the start of our study in 1992, and cohorts born from 1959 – 1983 were younger than age 60 years at the end of our study in 2018. For them, the FCP projects backward to age 60 for the older cohorts and forward to age 60 for the latter. Importantly, these projections are calculated using all of the model parameters which were estimated from all of the data.

The amount of extrapolation varies by birth year: 1 year *back* for the 1931 cohort, 1 year *forward* for the 1959 cohort, 2 years *back* for 1930 cohort, 2 years *forward* for the 1960 cohort, etc.

Therefore, in our analysis, we are incorporating from 1 - 6 years of forward extrapolation for the Baby Boomer cohorts born from 1959 - 1964, and from 7 - 22 years of forward extrapolation for Generation X cohorts born from 1965 - 1980.

It is appropriate to interpret the FCP when the model fit is adequate. That is why this Supplement contains a detailed analysis of LOF.

© 2024 Rosenberg PS et al. *JAMA Network Open*.

SEER Data

Our study complies with the principles of the STROBE statement for observational studies. SEER data are ascertained year-by-year (cross-sectionally), but can be analyed using both cross-sectional and longitudinal statistical methods.

eTable 1. Organ-Specific Site Code Used in the Classification of Cancer Sites Using SEER*Stat 8.4.0

Cancer site	Selection statement
**Esophagus**	{Site and Morphology.Primary Site - labeled} = 'C15.0-Cervical esophagus','C15.1-Thoracic esophagus','C15.2-Abdominal esophagus','C15.3-Upper third of esophagus','C15.4-Middle third of esophagus','C15.5-Lower third of esophagus','C15.8-Overlapping lesion of esophagus','C15.9-Esophagus, NOS'
**Stomach**	{Site and Morphology.Primary Site - labeled} = 'C16.0-Cardia, NOS','C16.1-Fundus of stomach','C16.2-Body of stomach','C16.3-Gastric antrum','C16.4-Pylorus','C16.5-Lesser curvature of stomach NOS','C16.6-Greater curvature of stomach NOS','C16.8-Overlapping lesion of stomach','C16.9-Stomach, NOS'
**Colon**	{Site and Morphology.Primary Site - labeled} = 'C18.0-Cecum','C18.1-Appendix','C18.2-Ascending colon','C18.3-Hepatic flexure of colon','C18.4-Transverse colon','C18.5-Splenic flexure of colon','C18.6-Descending colon','C18.7-Sigmoid colon','C18.8-Overlapping lesion of colon','C18.9-Colon, NOS'
**Rectum**	{Site and Morphology.Primary Site - labeled} = 'C20.9-Rectum, NOS'
**Liver**	{Site and Morphology.Primary Site - labeled} = 'C22.0-Liver'
**Gallbladder**	{Site and Morphology.Primary Site - labeled} = 'C23.9-Gallbladder'
**Pancreas**	{Site and Morphology.Primary Site - labeled} = 'C25.0-Head of pancreas','C25.1-Body of pancreas','C25.2-Tail of pancreas','C25.3-Pancreatic duct','C25.4-Islets of Langerhans','C25.7-Other specified parts of pancreas','C25.8-Overlapping lesion of pancreas','C25.9-Pancreas, NOS'
**Lung and Bronchus**	{Site and Morphology.Primary Site - labeled} = 'C34.0-Main bronchus','C34.1-Upper lobe, lung','C34.2-Middle lobe, lung','C34.3-Lower lobe, lung','C34.8-Overlapping lesion of lung','C34.9-Lung, NOS'
**Melanoma of skin**	{Site and Morphology.Primary Site - labeled} = 'C44.0-Skin of lip, NOS','C44.1-Eyelid','C44.2-External ear','C44.3-Skin other/unspec parts of face','C44.4-Skin of scalp and neck','C44.5-Skin of trunk','C44.6-Skin of upper limb and shoulder','C44.7-Skin of lower limb and hip','C44.8-Overlapping lesion of skin','C44.9-Skin, NOS'
**Breast**	{Site and Morphology.Primary Site - labeled} = 'C50.0-Nipple','C50.1-Central portion of breast','C50.2-Upper-inner quadrant of breast','C50.3-Lower-inner quadrant of breast','C50.4-Upper-outer quadrant of breast','C50.5-Lower-outer quadrant of breast','C50.6-Axillary tail of breast','C50.8-Overlapping lesion of breast','C50.9-Breast, NOS'
**Cervix uterus**	{Site and Morphology.Primary Site - labeled} = 'C53.9-Cervix uteri'
**Corpus uterus**	{Site and Morphology.Primary Site - labeled} = 'C54.9-Corpus uteri'
**Ovary**	{Site and Morphology.Primary Site - labeled} = 'C56.9-Ovary'
**Prostate gland**	AND {Site and Morphology.Primary Site - labeled} = 'C61.9-Prostate gland'
**Kidney**	{Site and Morphology.Primary Site - labeled} = 'C64.9-Kidney, NOS','C65.9-Renal pelvis'
**Urinary Bladder**	{Site and Morphology.Primary Site - labeled} = 'C67.0-Trigone of bladder','C67.1-Dome of bladder','C67.2-Lateral wall of bladder','C67.3-Anterior wall of bladder','C67.4-Posterior wall of bladder','C67.5-Bladder neck','C67.6-Ureteric orifice','C67.7-Urachus','C67.8-Overlapping lesion of bladder','C67.9-Bladder, NOS'
**Brain and CNS**	{Site and Morphology.Primary Site - labeled} = 'C71.0-Cerebrum','C71.1-Frontal lobe','C71.2-Temporal lobe','C71.3-Parietal lobe','C71.4-Occipital lobe','C71.5-Ventricle, NOS','C71.6-Cerebellum, NOS','C71.7-Brain stem','C71.8-Overlapping lesion of brain','C71.9-Brain, NOS','C72.0-Spinal cord','C72.1-Cauda equina','C72.2-Olfactory nerve','C72.3-Optic nerve','C72.4-Acoustic nerve','C72.5-Cranial nerve, NOS','C72.8-Overlapping lesion of brain & CNS','C72.9-Nervous system, NOS'
**Thyroid**	{Site and Morphology.Primary Site - labeled} = 'C73.9-Thyroid gland'
**Non-Hodgkin Lymphoma**	AND {Site and Morphology.Site recode ICD-O-3/WHO 2008} = ' Non-Hodgkin Lymphoma',' NHL - Nodal',' NHL - Extranodal'
**Multiple Myeloma**	{Site and Morphology.Site recode ICD-O-3/WHO 2008} = 'Myeloma'<br>AND {Site and Morphology.ICD-O-3 Hist/behavior, labeled} = '9732/3: Plasma cell (multiple) myeloma'
**Leukemia**	AND {Site and Morphology.Site recode ICD-O-3/WHO 2008} = ' Leukemia',' Lymphocytic Leukemia',' Acute Lymphocytic Leukemia',' Chronic Lymphocytic Leukemia',' Other Lymphocytic Leukemia'

© 2024 Rosenberg PS et al. *JAMA Network Open*.

eTable 2. **Age-Standardized Incidence Rates and Cancer Cases by Sex, Race and Ethnicity, United States, 35-84 Years Old, 1992-2018**

*ASR – Age Standardize Rate (US 2000 Standard Population).

Registries included in SEER13: the Alaska Native, Atlanta, Connecticut, Detroit, Hawaii, Iowa, Los Angeles, New Mexico, Rural Georgia, San Francisco-Oakland, San Jose-Monterey, Seattle-Puget Sound, and Utah.

	Non-Hispanic White		Non-Hispanic Black		Hispanic		Asian or Pacific Islander	
	ASR*	cases	ASR	cases	ASR	cases	ASR	cases
**Male**								
Esophagus	14.5	23862	16.6	3388	9.3	2541	7.8	2124
Stomach	15.7	25169	28.7	5427	26.8	7297	30.4	8001
Colon	63.9	102017	85.3	16393	51.5	13968	54.1	14555
Rectum	21.3	34790	22.9	4758	20.4	6094	23.8	6724
Liver	13.4	22514	29.1	6399	31.3	9317	37.1	10389
Gallbladder	1.2	1854	1.9	351	2.2	563	1.9	491
Pancreas	24.3	39312	32.5	6351	20.6	5467	19.4	5143
Lung and Bronchus	131.4	211745	182.7	35125	69.6	16872	96.4	25326
Melanoma of the Skin	61.9	100007	2.1	414	8.5	2524	2.7	759
Breast	2.2	3512	3.2	654	1.1	314	1.1	303
Prostate	285.9	474797	457.1	92109	219.6	57321	159	42171
Urinary Bladder	71.1	113074	37.3	6780	32.2	7875	28.6	7363
Kidney and Renal Pelvis	35	57455	44.2	9289	34.5	10416	21.3	5950
Brain and CNS	13.6	21998	7.2	1524	8.9	2873	5.9	1642
Thyroid	10.6	17234	5.7	1254	7.7	2719	9.4	2733
Non-Hodgkin Lymphoma	44.9	71997	33.7	7215	34.8	10392	29	7865
Myeloma	13.3	21478	28.6	5547	12.9	3502	8.4	2247
Leukemia	30.9	49442	23.4	4565	17.8	5172	14.8	3972
**Female**								
Esophagus	3.5	6664	5.7	1513	1.8	570	1.8	589
Stomach	6.7	12739	14.6	3718	16.3	5613	16.6	5513
Colon	51.2	97322	68.1	17672	39.2	13165	41	13769
Rectum	13	24053	14.6	3946	11.5	4147	13.3	4539
Liver	3.8	7219	7.8	2109	10.3	3330	12.1	4044
Gallbladder	2	3801	3.2	822	5.8	1939	2.6	858
Pancreas	18	34476	26.4	6745	17.4	5581	15.4	5093
Lung and Bronchus	98.5	188028	100	26210	43.4	13922	52.4	17561
Melanoma of the Skin	38.5	66649	1.4	371	8.1	3073	2.1	726
Breast	265.3	478203	239	65710	177.4	67233	196.5	67995
Cervix Uteri	11.1	18177	17.1	4756	21.3	8842	14.1	4826
Corpus Uteri	51.8	96494	42.6	11676	38.6	14593	38.9	13635
Ovary	25.3	46112	18.1	4884	20.2	7443	18.1	6221
Urinary Bladder	17.6	33632	12.5	3122	8.7	2744	7	2327
Kidney and Renal Pelvis	16.6	30732	20.6	5490	19.3	6882	9.6	3280
Brain and CNS	9.1	16372	4.9	1313	6.5	2404	4.1	1390
Thyroid	25.5	42044	16.8	4742	25.3	10455	27.2	9393
Non-Hodgkin Lymphoma	30.5	56766	21.9	5867	26.3	9055	19.8	6669
Myeloma	8.2	15504	21.2	5515	9	3015	5.8	1952
Leukemia	17.4	32641	14.1	3679	12	4222	9	3038

Because SAGE analysis is carried out on the log scale, we added 0.5 to the event count for any age- and period-specific cell with 0 observed events.

© 2024 Rosenberg PS et al. *JAMA Network Open*.

Supplemental Figures

eFigure 1. Observed Rates in Females

Lexis diagrams by cancer site, race and ethnicity: Females. Heat Maps show observed incidence by single-years of age (35 - 84 years from bottom to top) and single calendar years (1992 - 2018 from left to right). Birth cohorts track along diagonals (lower left to upper right) within each panel. Values within each Heat Map are divided by the corresponding maximum value. Values from 0 through 1 are color-coded using the colorbar located on the right hand side of the panel array. Jet colormap: blue (lowest) - green - yellow - orange - red (highest). Cancer sites along the rows. Race and ethnicity groups down the columns.

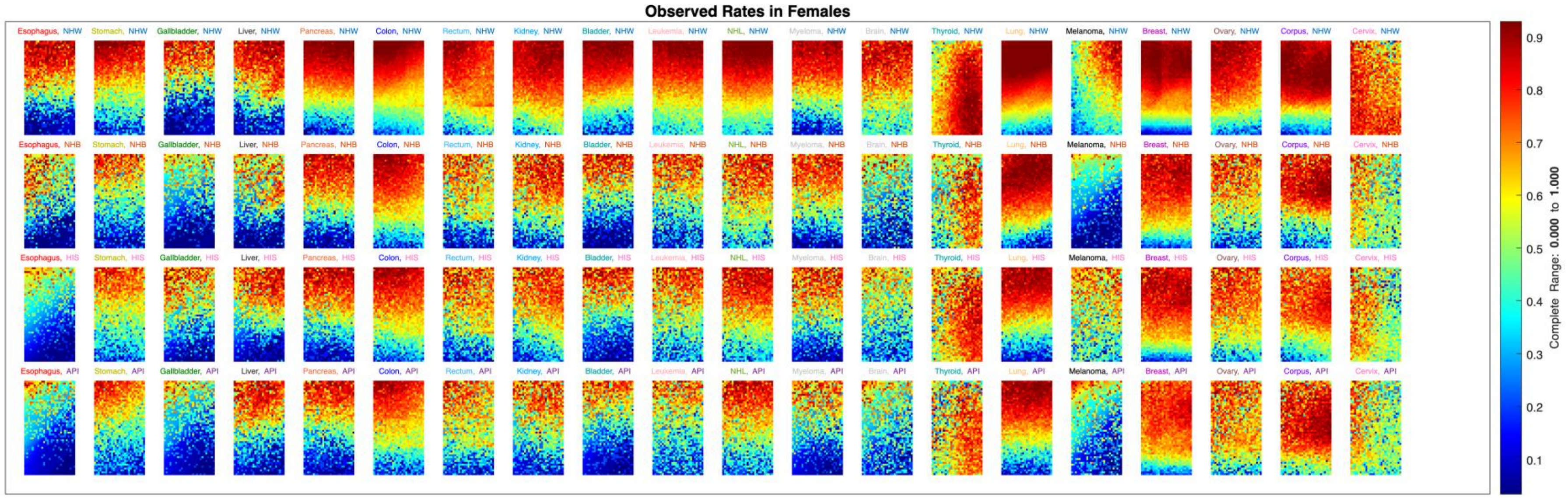

© 2024 Rosenberg PS et al. *JAMA Network Open*.

eFigure 2. APC Fitted Values in Females

Lexis diagrams of fitted rates obtained from semi-parametric age-period-cohort (SAGE) analysis: Females. See Legend to eFigure 1 for details.

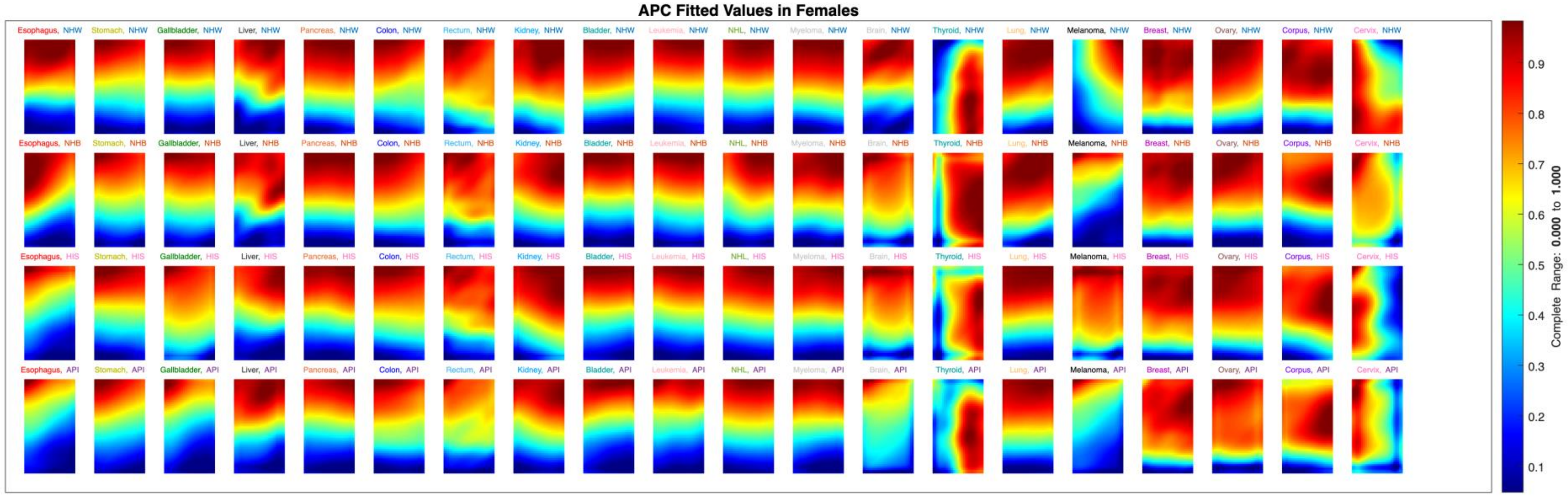

© 2024 Rosenberg PS et al. *JAMA Network Open*.

eFigure 3. Observed Rates in Males

Lexis diagrams by cancer site, race and ethnicity: Males. See Legend to eFigure 1 for details.

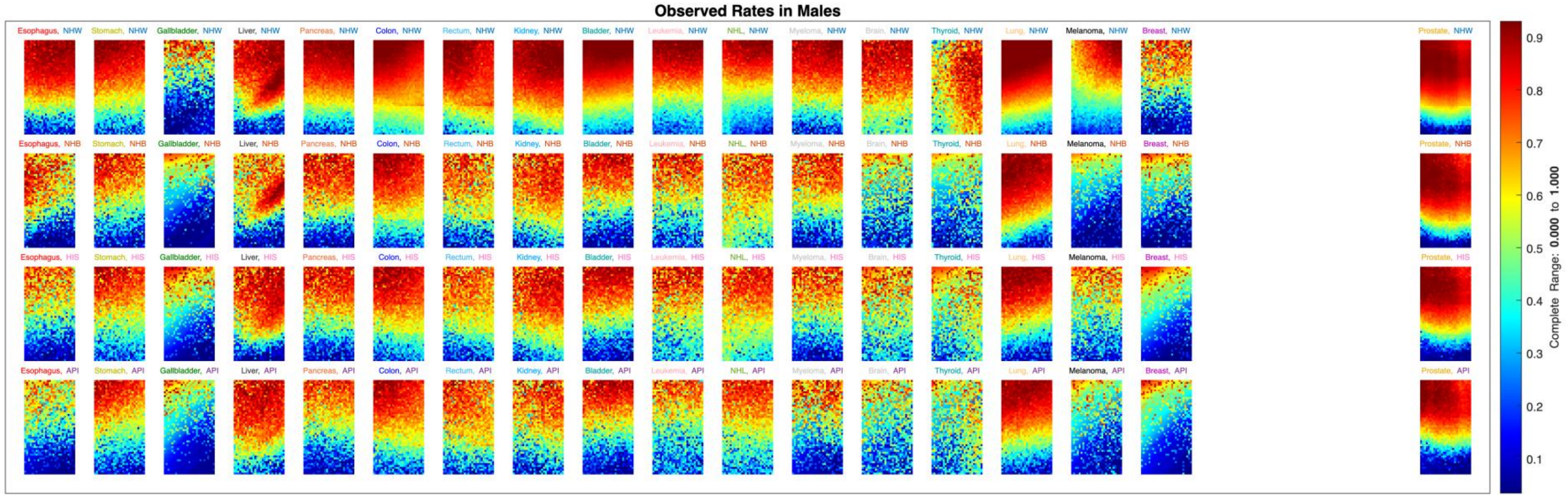

© 2024 Rosenberg PS et al. *JAMA Network Open*.

eFigure 4. APC Fitted Values in Males

Lexis diagrams of fitted rates obtained from semi-parametric age-period-cohort (SAGE) analysis: Males. See Legend to eFigure 1 for details.

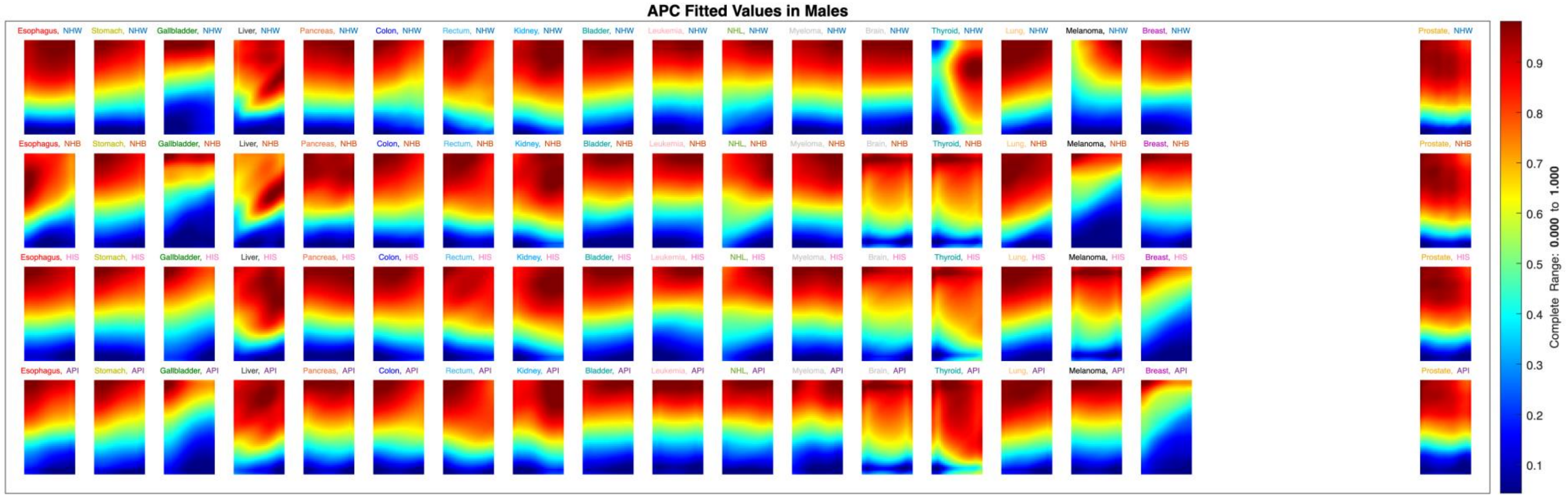

© 2024 Rosenberg PS et al. *JAMA Network Open*.

Assessment of Lack-of-Fit (LOF):

eFigure 5. Lack of Fit (LOF) in Females

LOF corresponding to APC Fitted Values shown in eFigure 2: Females. See Statistical Methods details.

Heat Maps of residuals by single-years of age (35 - 84 years from bottom to top) and single calendar years (1992 - 2018 from left to right). Values from -0.3 to +0.3 are color-coded using the colorbar located on the right hand side of the panel array. Green-to-Red colormap: green (lowest) - black (zero) - red (highest).

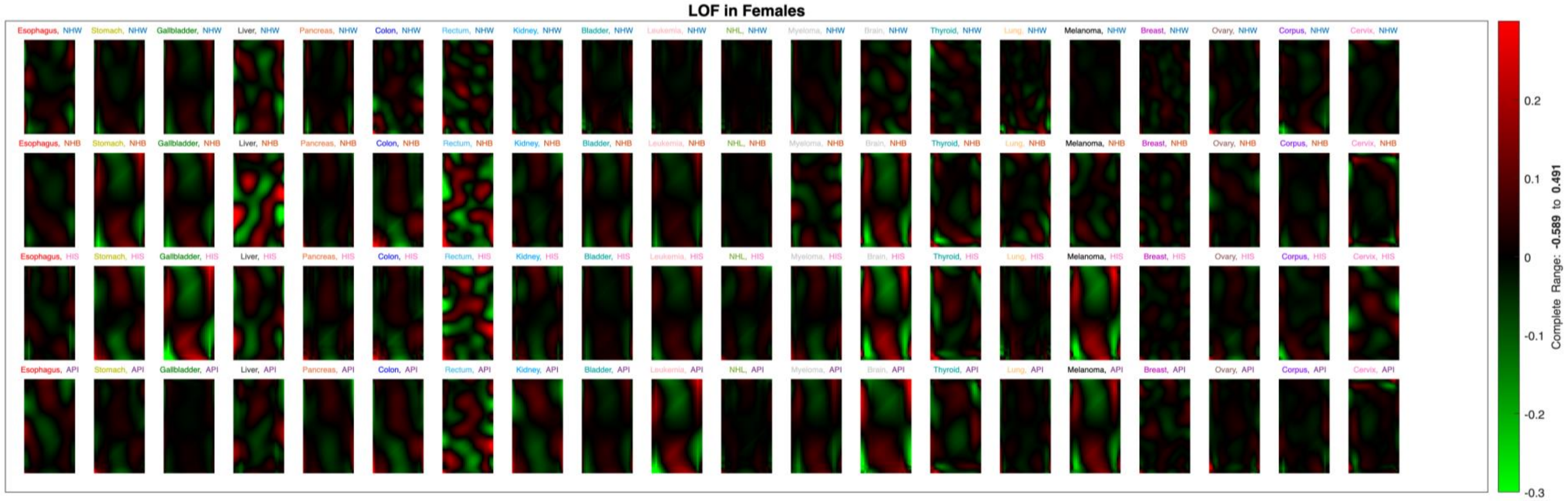

© 2024 Rosenberg PS et al. *JAMA Network Open*.

eFigure 6. Lack of Fit (LOF) in Males

LOF corresponding to APC Fitted Values shown in eFigure 4: Males. See Legend to eFigure 5 for details.

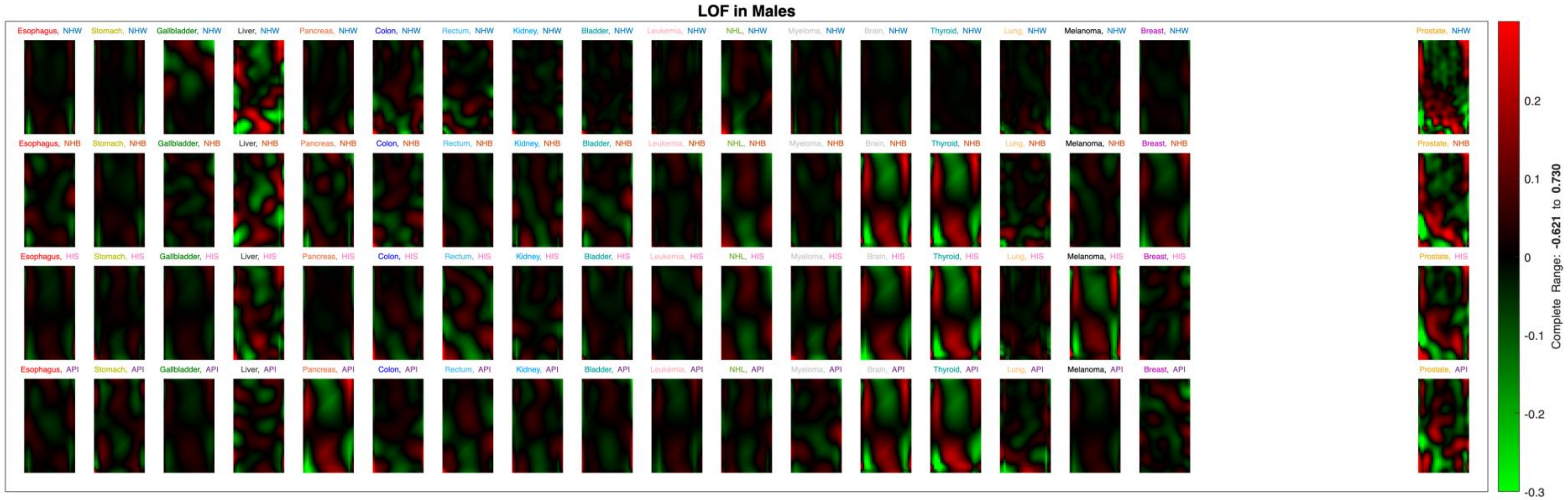

© 2024 Rosenberg PS et al. *JAMA Network Open*.

eFigure 7. Higher-Order Deviations vs Lack of Fit (LOF) in Females

Circular Heat Maps compare Higher-Order Deviations (outer ring) to the corresponding LOF (inner ring) by cancer site, race, and ethnicity in Females. All panels are plotted using the same green-to-red colormap: green (lowest) - black (zero) - red (highest). LOF is generally small compared to the corresponding Higher-Order Deviations.

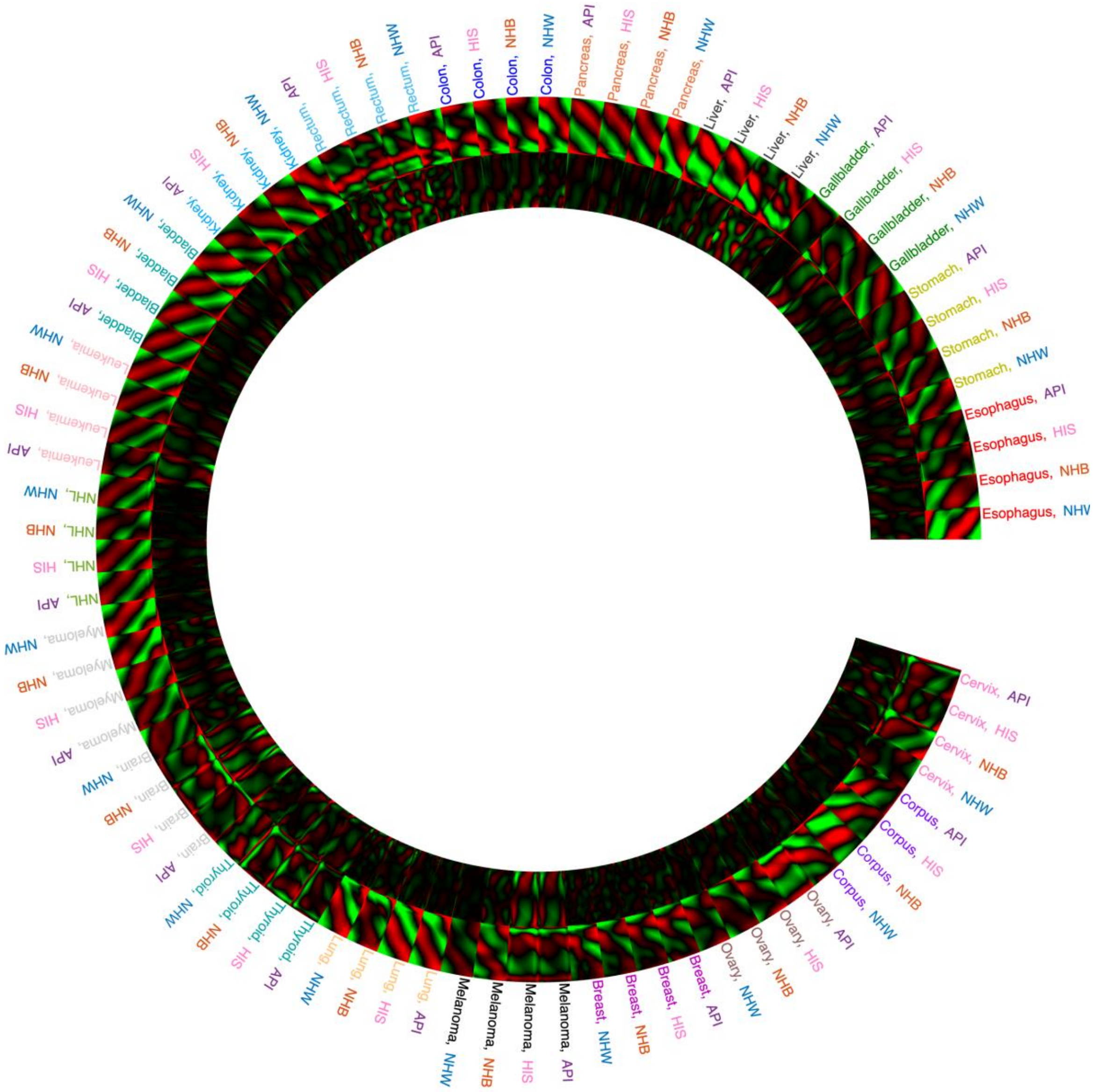

© 2024 Rosenberg PS et al. *JAMA Network Open*.

eFigure 8. Higher-Order Deviations vs Lack of Fit (LOF) in Males

See Legend to eFigure 7 for details.

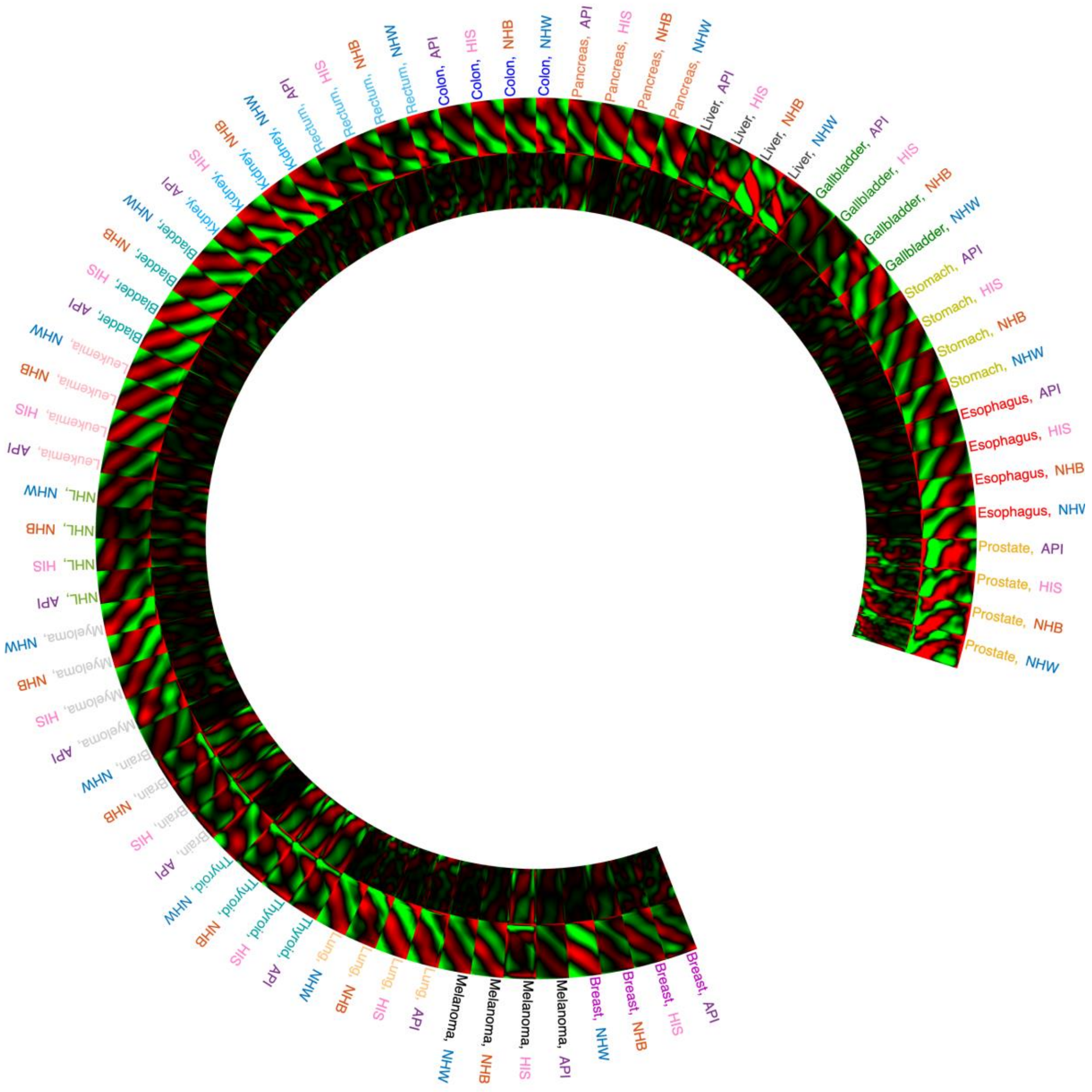

© 2024 Rosenberg PS et al. *JAMA Network Open*.

eFigure 9. Local Drifts in Females

Local Drifts: Estimated Annual Percentage Change (EAPC) over time conditional on age: Females. Panels: Female cancer sites. Curves within panels: NHW (solid blue); NHB (dot-dash red); HIS (dash gold); API (dotted magenta).

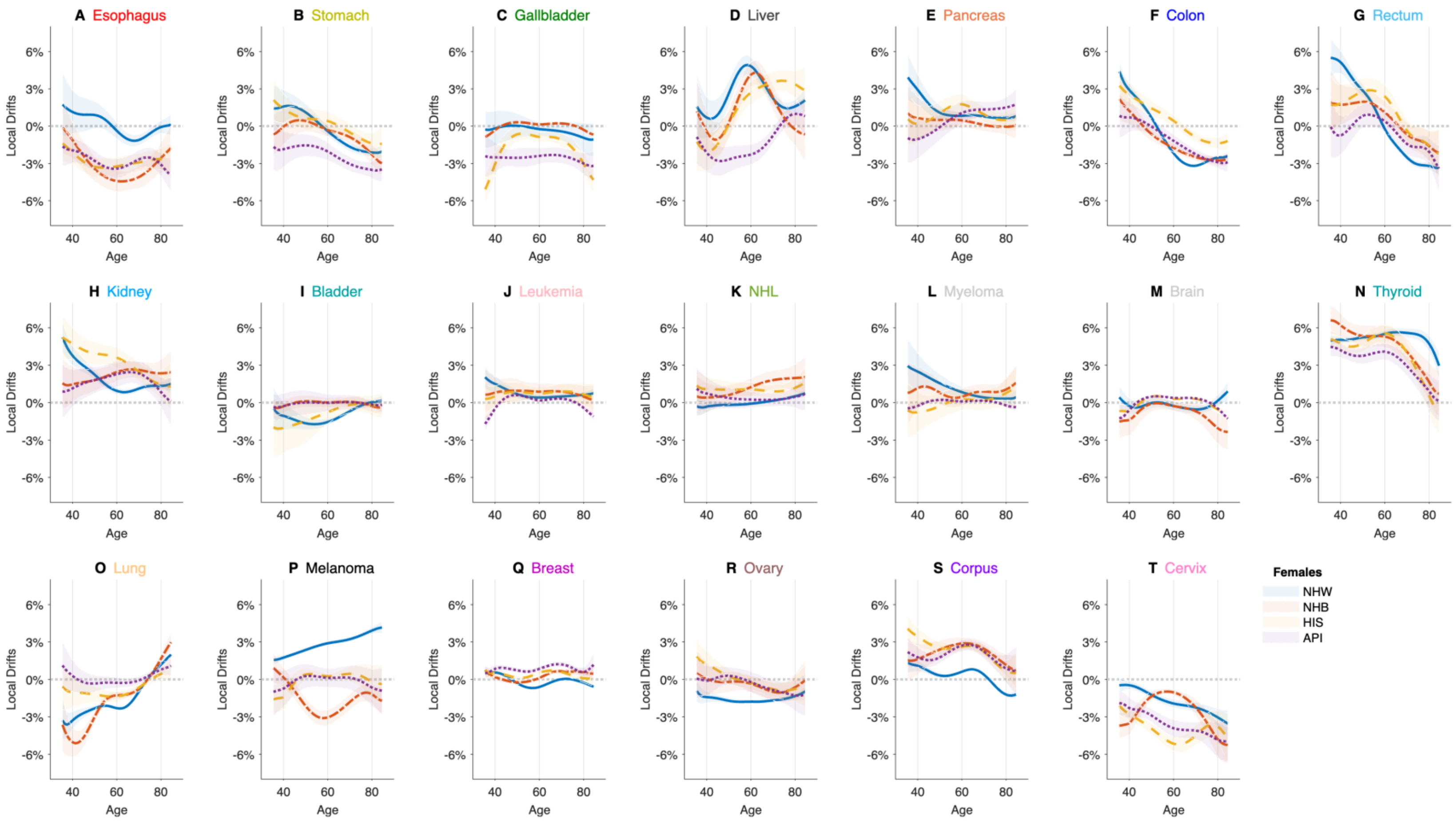

© 2024 Rosenberg PS et al. *JAMA Network Open*.

eFigure 10. Local Drifts in Males

Local Drifts: Estimated Annual Percentage Change (EAPC) over time conditional on age: Males. Panels: Male cancer sites. Curves within panels: NHW (solid blue); NHB (dot-dash red); HIS (dash gold); API (dotted magenta).

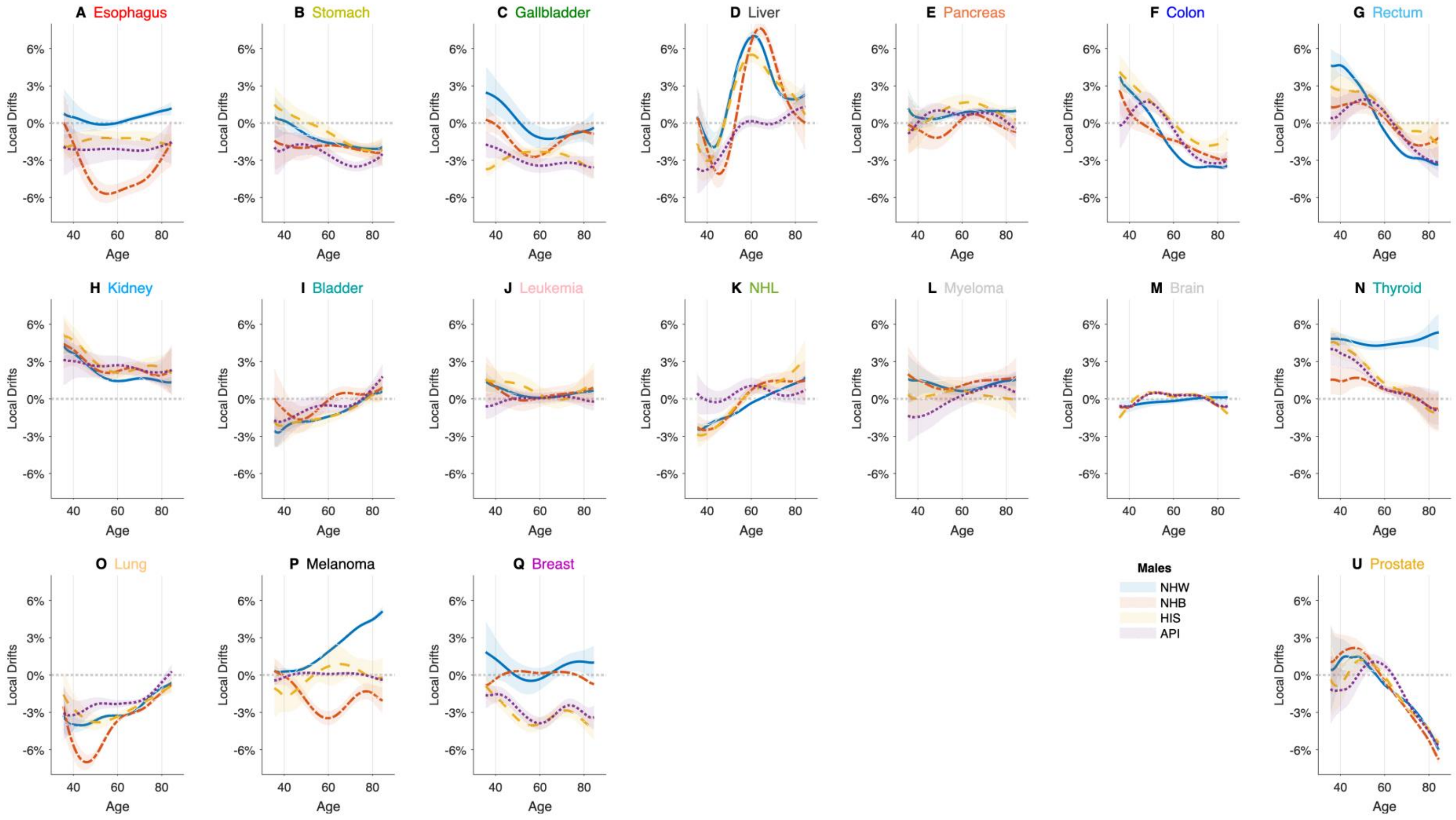

© 2024 Rosenberg PS et al. *JAMA Network Open*.

eFigure 11. Fitted Cohort Patterns (FCPs) by Cancer Site, Race, and Ethnicity: Females

FCP, estimated incidence per 100,000 person-years at age 60 years by birth year (1908 – 1983). All curves on the log10 scale. FCPs by cancer site in panels **A**– **T.** Within panels: NHW (solid blue), NHB (dot-dash red), HIS (dash gold), and API (dot magenta). Tick marks on x-axes indicate start years for consecutive social generations: 1928 – Silent; 1946 – Baby Boomers; 1965 – Generation X.

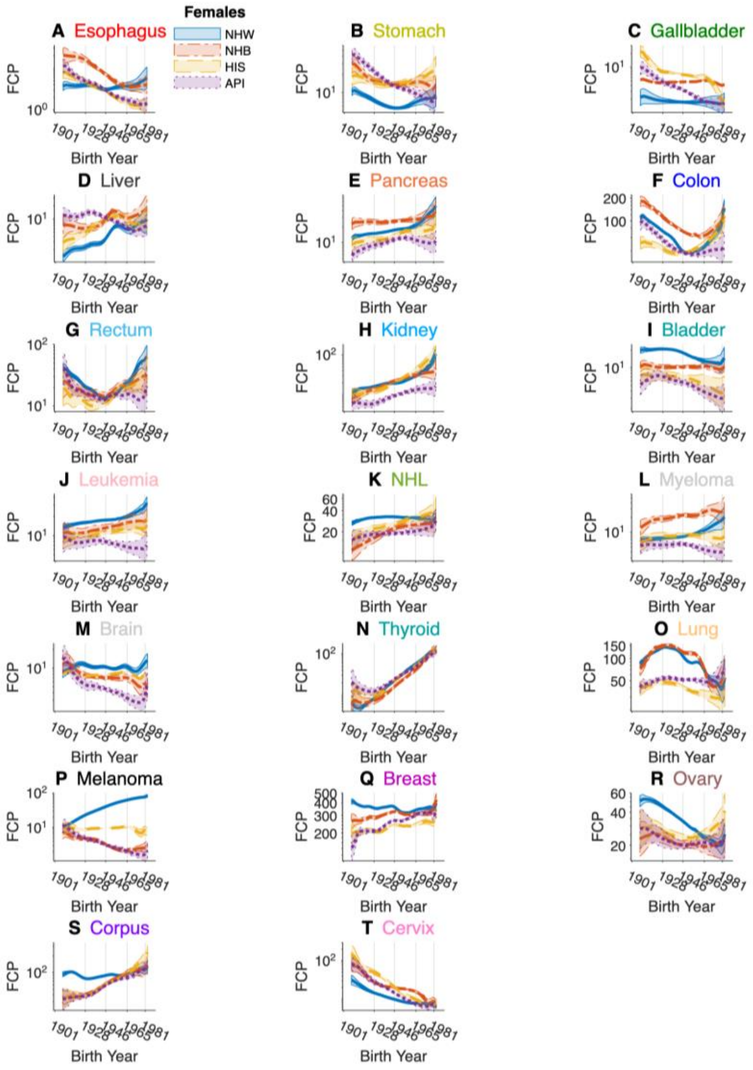

© 2024 Rosenberg PS et al. *JAMA Network Open*.

eFigure 12. Estimated Annual Percentage Change (EAPC) of the Fitted Cohort Pattern (FCP): Females

We fitted a JoinPoint model to each FCP curve presented in eFigure 11, accounting for the estimated variance-covariance matrix of the FCP and allowing for up to 5 segments each with 10 or more birth years. We estimated the best fit model using the JoinPoint permutation test. Heat Map shows best-fit EAPC values by birth year, with rows corresponding to cancer site and race and ethnicity within cancer site, as indicated in the y-axis labels. Birth years within social generations are color coded within the x-axis labels: Greatest (1908 - 1927); Silent (1928 - 1945); Boomer (1946 - 1964); Generation X (1965 - 1980); Millennials (1981 - 1983). Values from -6%/year to +6%/year are color-coded using the colorbar located on the right hand side of the panel array: green (lowest) - black (zero) - red (highest).

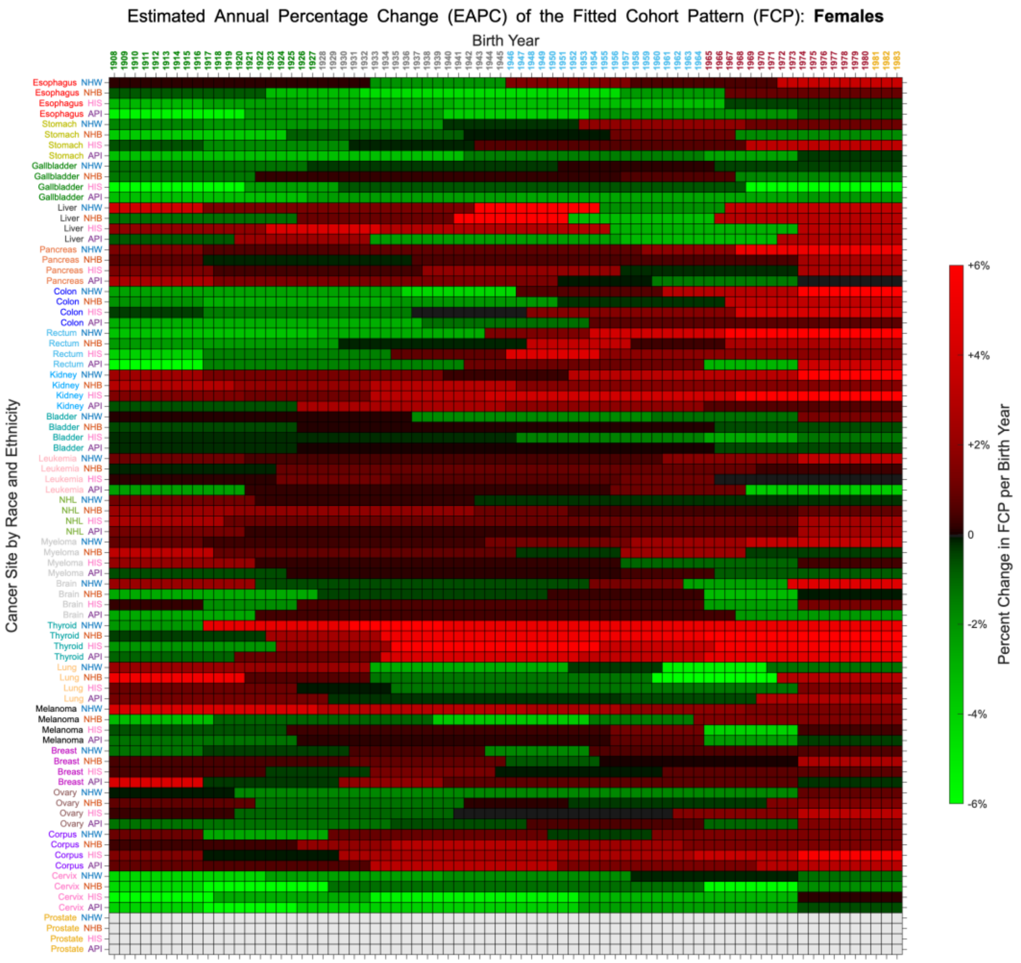

© 2024 Rosenberg PS et al. *JAMA Network Open*.

eFigure 13. Fitted Cohort Patterns (FCPs) by Cancer Site, Race, and Ethnicity: Males

See legend to eFigure 11 for details.

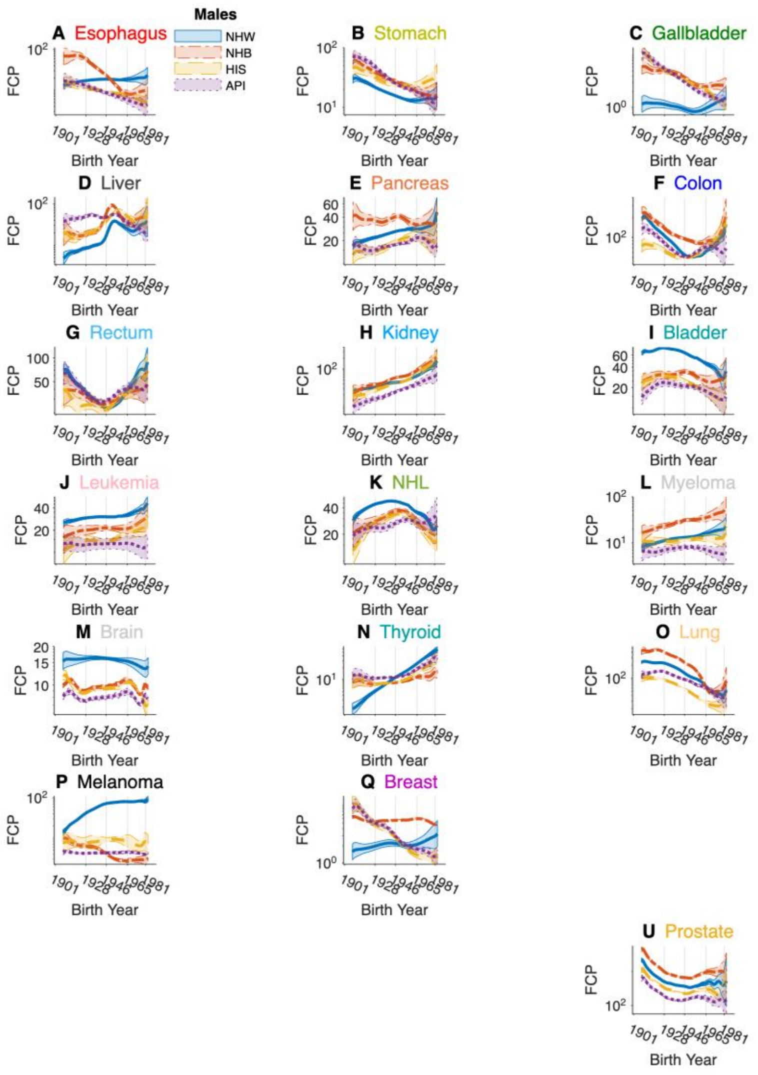

© 2024 Rosenberg PS et al. *JAMA Network Open*.

eFigure 14. Estimated Annual Percentage Change (EAPC) of the Fitted Cohort Pattern (FCP): Males

We fitted a JoinPoint model to each FCP curve presented in eFigure 13. See Legend to eFigure 12 for details.

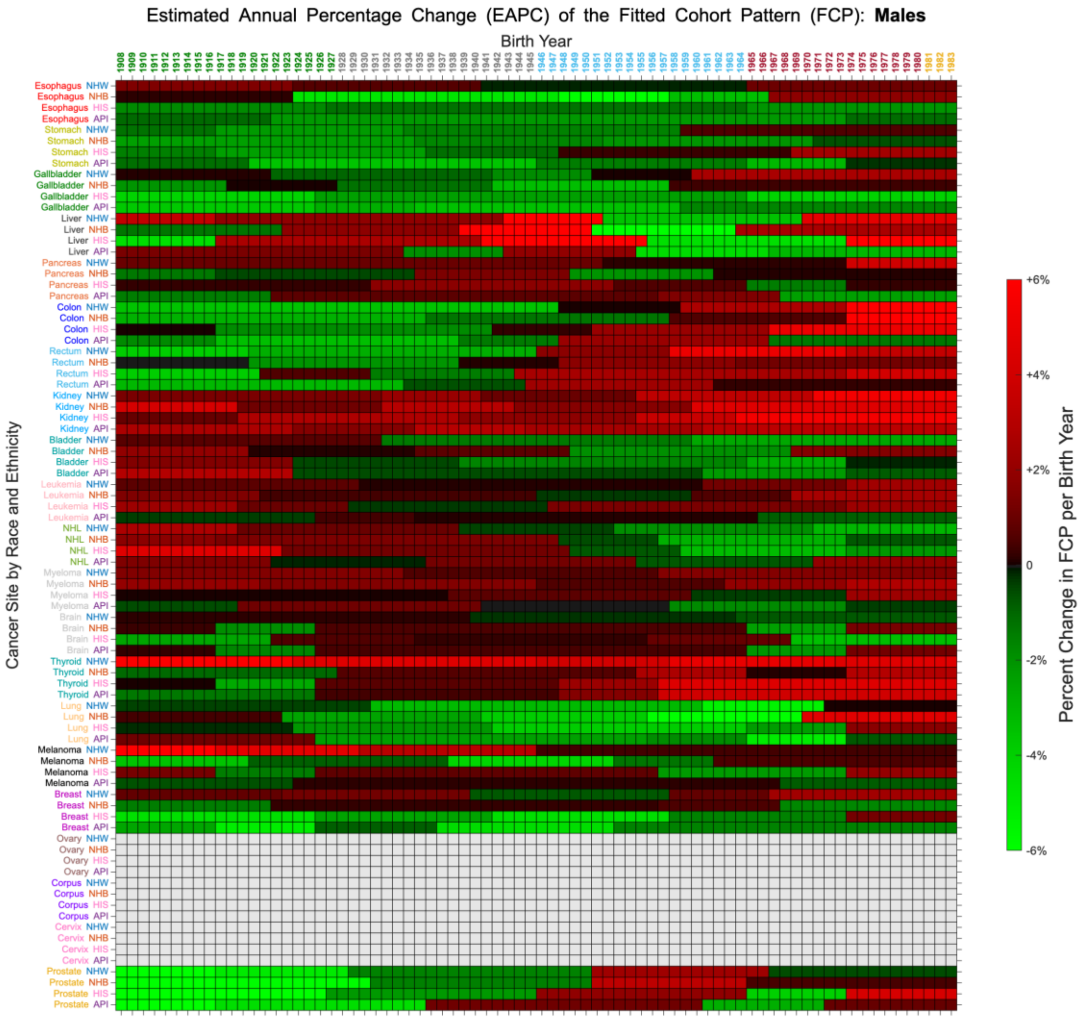

© 2024 Rosenberg PS et al. *JAMA Network Open*.

eFigure 15. Average Incidence at Age 60: Generation X vs Baby Boomers

The Fitted Cohort Pattern (FCP) describes the absolute incidence of a cancer at an arbitrary reference age (here, age 60 years) for each birth year in the Lexis diagram (1908 - 1983). FCP values can be averaged within social generations. Arrow Plots indicate magnitude and direction of change from Baby Boomers born 1946 - 1964 (blue dots) to Generation X born 1965 - 1980 (yellow triangles). Panels **A** - **H** show average FCP values (rates per 100,000 persons per year) by site conditional on sex, race, and ethnicity. Arrows are plotted on the natural log scale. Text annotations give corresponding FCP values on the absolute scale. Within each panel, sites are sorted in order of relative change, from largest relative increase (top) to largest relative decrease (bottom).

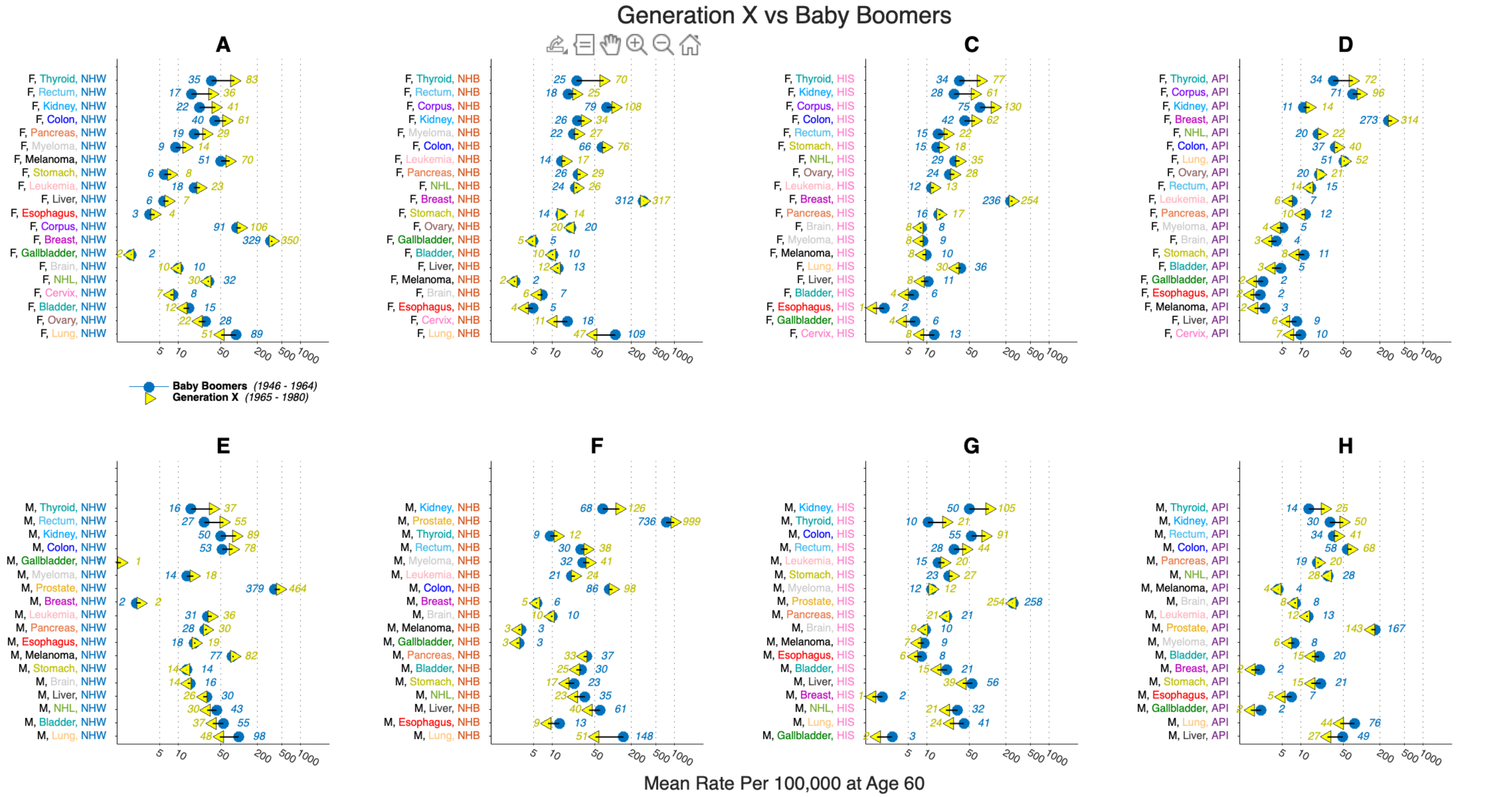

© 2024 Rosenberg PS et al. *JAMA Network Open*.

eFigure 16. Average Incidence at Age 60: Baby Boomers vs the Silent Generation

See Legend to eFigure 15 for details.

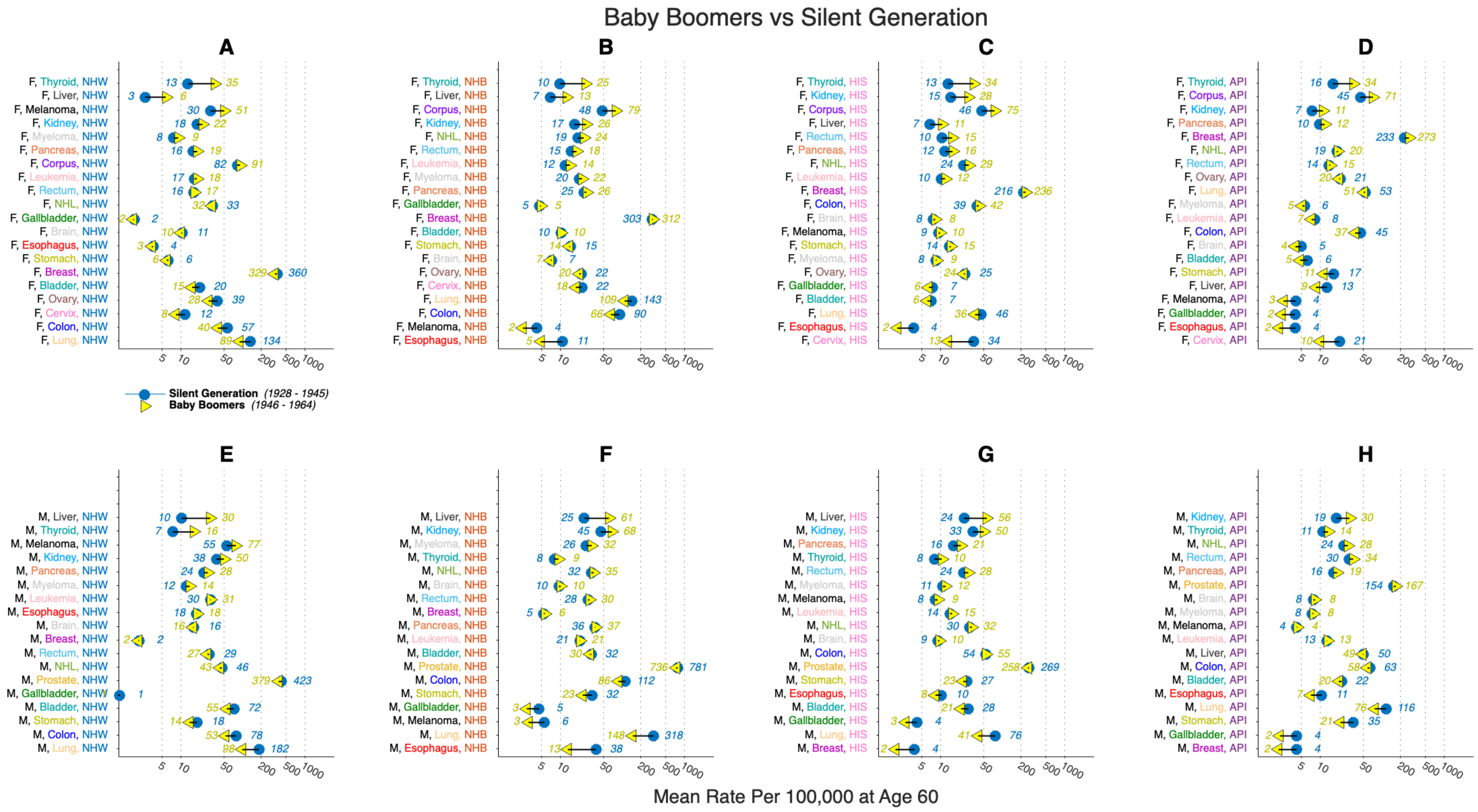

© 2024 Rosenberg PS et al. *JAMA Network Open*.

eFigure 17. Average Incidence at Age 60: Silent vs Greatest Generations

See Legend to eFigure 15 for details.

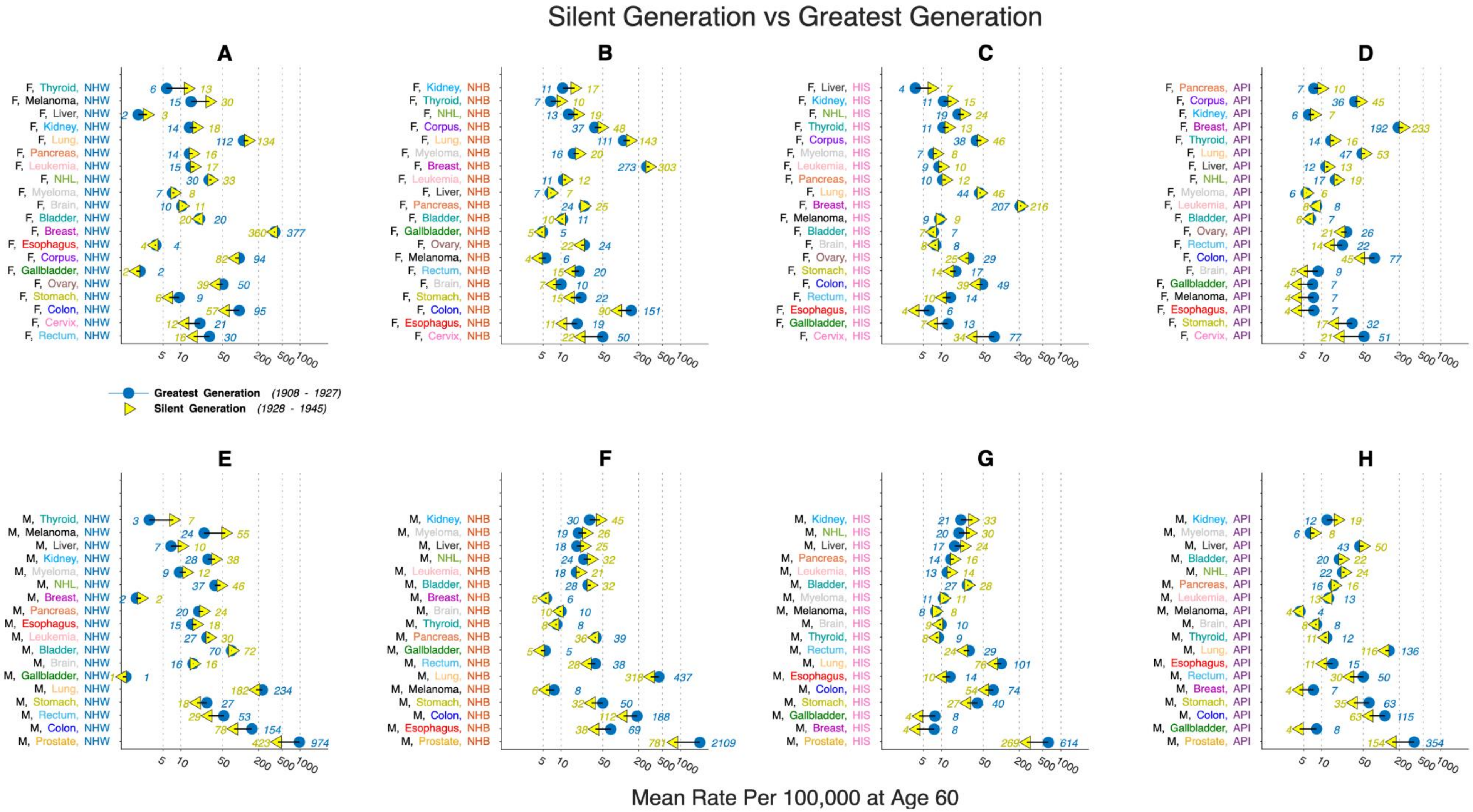

© 2024 Rosenberg PS et al. *JAMA Network Open*.

eFigure 18. Site-Adjusted Cancer Incidence Rate Ratios (IRRs) for Non-Hispanic Black, Hispanic, and Asian or Pacific Islander vs Non-Hispanic White by Sex and Social Generation

Race and ethnicity contrasts corresponding to the site-specific IRRs shown in panels A - F in Main Figure 4.

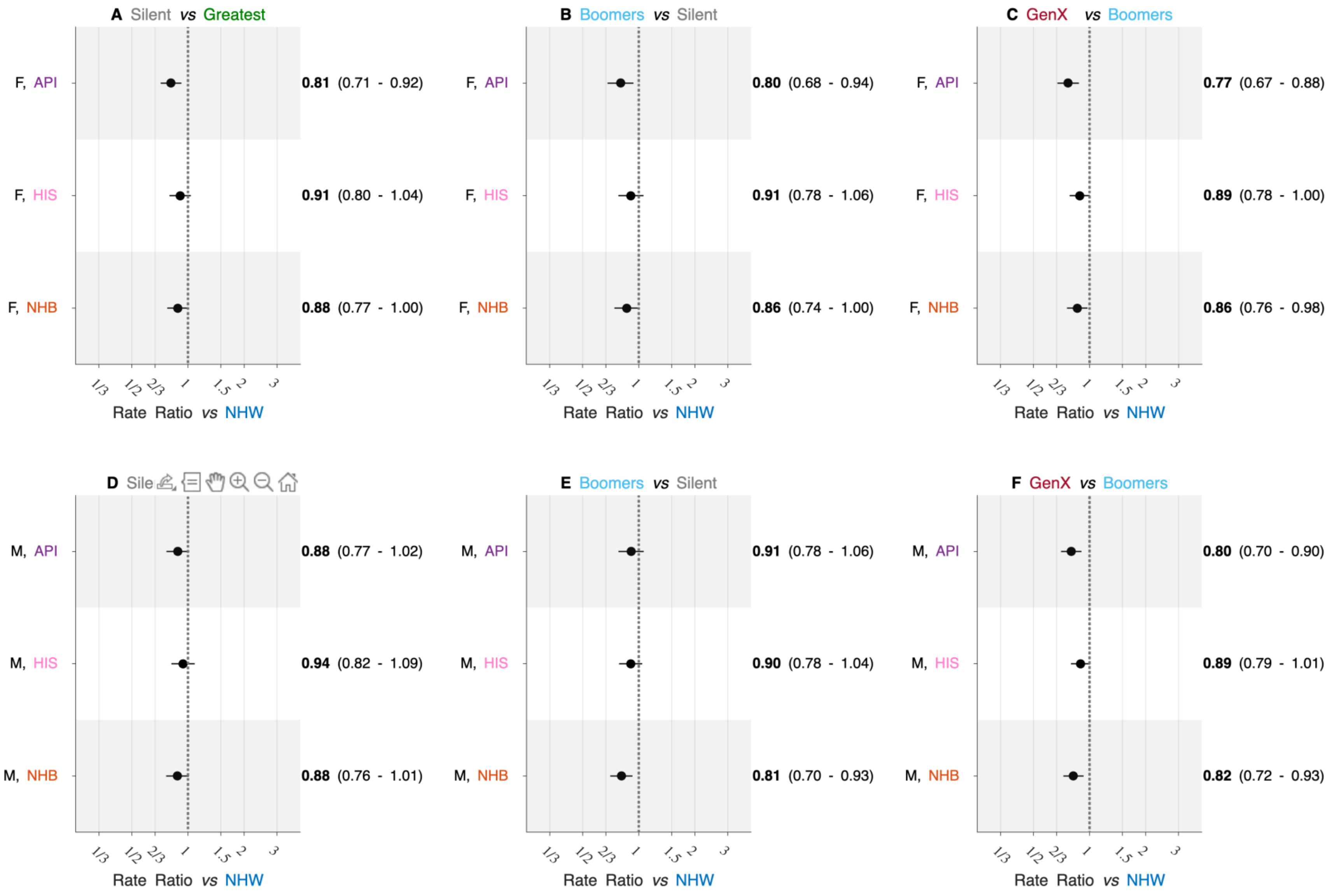

© 2024 Rosenberg PS et al. *JAMA Network Open*.

eFigure 19. Percent Changes in Incidence of Leading Cancers at Age 60 Years per 100,000 Person-Years in Successive Generations

See legend to main Figure 5 for details.

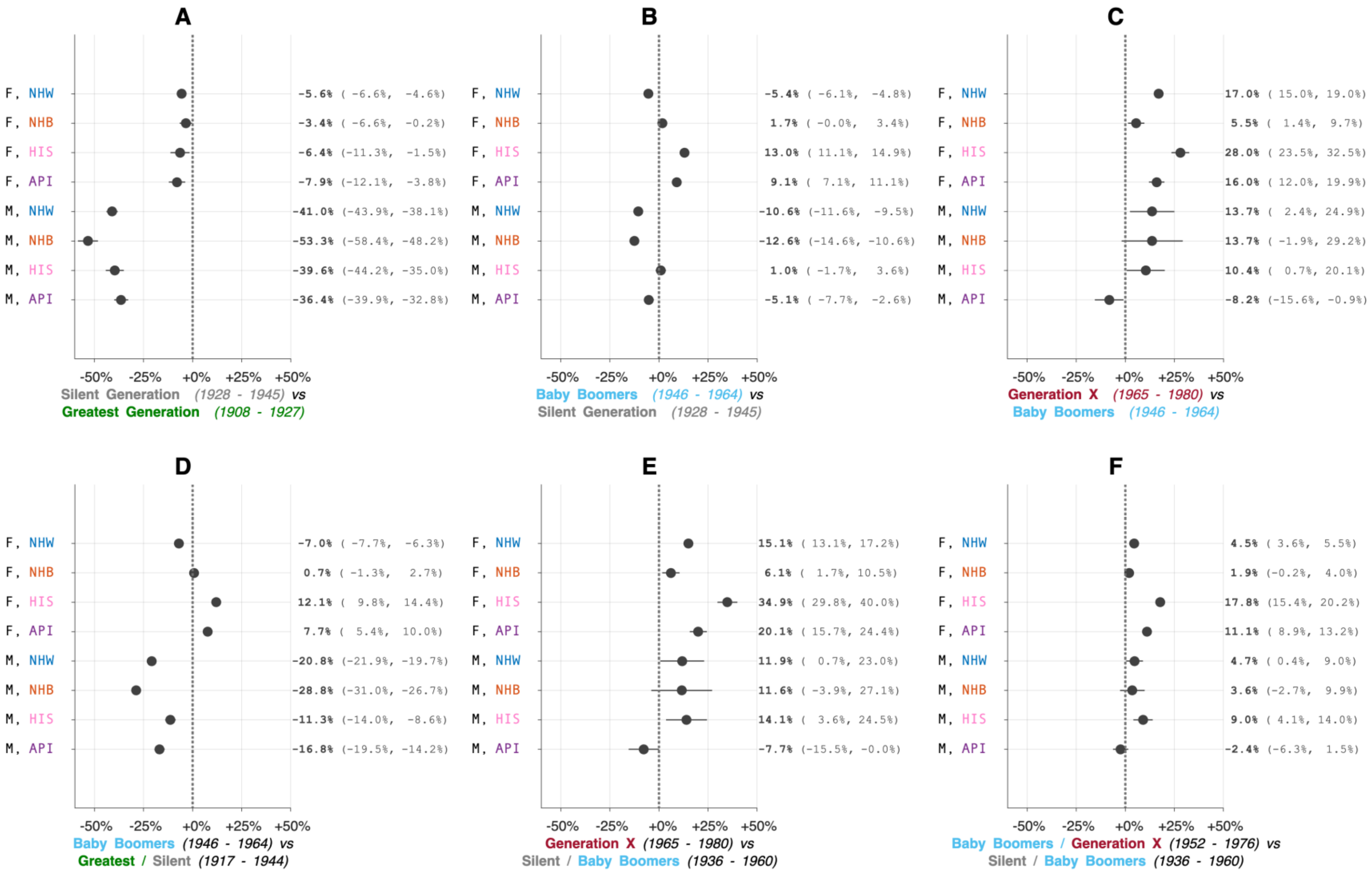

© 2024 Rosenberg PS et al. *JAMA Network Open*.
