# Supplementary material for: Cancer Incidence Trends in Successive Social Generations in the US
Source: JAMA Netw Open. 2024 Jun 10;7(6):e2415731. doi: 10.1001/jamanetworkopen.2024.15731 (PMC11165384; doi:10.1001/jamanetworkopen.2024.15731)
Supplement: Supplement 2. — Data Sharing Statement [file jamanetwopen-e2415731-s002.pdf]

Data Sharing Statement

Rosenberg. Cancer Incidence Trends in Successive Social Generations in the US. *JAMA Netw Open*. Published June 10, 2024. doi:10.1001/jamanetworkopen.2024.15731

Data
Data available: Yes
Data types: Data (not involving human participants), Data dictionary
How to access data: See https://seer.cancer.gov/data/
When available: With publication

Supporting Documents
Document types: None

Additional Information
Who can access the data: anyone requesting the data
Types of analyses: any purpose
Mechanisms of data availability: with investigator support
